# Identifying P100 and N170 as electrophysiological markers for conscious and unconscious processing of emotional facial expressions

**DOI:** 10.3389/fnbeh.2024.1464888

**Published:** 2025-01-23

**Authors:** Lennard Herzberg, Julia Schräder, Han-Gue Jo, Ute Habel, Lisa Wagels

**Affiliations:** ^1^Department of Psychiatry, Psychotherapy and Psychosomatics, Faculty of Medicine, RWTH Aachen, Aachen, Germany; ^2^Institute of Neuroscience and Medicine, JARA-Institute Brain Structure Function Relationship (INM 10), Research Center Jülich, Jülich, Germany; ^3^Department of AI Convergence, College of Computer and Software, Kunsan National University, Gunsan, Republic of Korea

**Keywords:** event-related potentials, unconsciousness, emotion processing, P100, N170

## Abstract

**Introduction:**

Everyday life requires correct processing of emotions constantly, partly occurring unconsciously. This study aims to clarify the effect of emotion perception on different event-related potentials (ERP; P100, N170). The P100 and N170 are tested for their suitability as electrophysiological markers in unconscious processing.

**Methods:**

Using a modified backward masking paradigm, 52 healthy participants evaluated emotional facial expressions (happy, sad, or neutral) during EEG recording. While varying primer presentation time (16.7 ms for unconscious; 150 ms for conscious perception), either congruent or incongruent primer / target emotions were displayed.

**Results:**

The N170 was significantly larger in trials with conscious compared to unconscious primer presentation, while the P100 showed opposite results displaying higher amplitudes in unconscious versus conscious trials. The N170 amplitude was modulated by emotion.

**Discussion:**

Both P100 and N170 were modulated by stimulus presentation time, demonstrating the suitability as potential biomarkers and for systematic research on conscious and unconscious face processing.

## Introduction

Facial expressions are a vital part of non-verbal communication between humans and require fast and accurate cognitive processing (Batty and Taylor, 2003). Since the human capacity of processing information consciously is limited (Miller, 1956), some stimuli detected by the sensory system are perceived without awareness (Merikle et al., 2001). For clarity in our study, we specifically define “unconscious” processing as occurring when participants are exposed to a stimulus but lack conscious awareness of its specific content. This definition implies that while the stimulus is processed by the brain, participants do not have conscious access to the details of what was presented. By using this definition, we aim to distinguish our focus on processing that occurs below the threshold of conscious perception from related, yet distinct, concepts in the field.

Although highly relevant, many emotional stimuli are not consciously perceived and processed (Tamietto and de Gelder, 2010). Since especially emotional facial expressions as omnipresent stimuli of high relevance influence human behavior (Fox, 2002), understanding the mechanisms of early facial expression processing can provide a reference foundation for future research on unconscious emotion processing. Although brain anatomy and neural models of unconscious emotional processing have been discussed (for a review see Smith and Lane, 2016), the underlying electrophysiological correlates of unconscious emotional processing have yet to be explored further. Via a modified backward mask emotional conflict task, we specifically aim to establish suitable associative electrophysiological markers that can be used to differentiate conscious from unconscious emotion processing. Understanding these differences contributes to broader theories of emotional processing pathways, which suggest that unconscious perception may primarily rely on rapid subcortical pathways (Gainotti, 2012), whereas conscious perception engages more detailed cortical processing, particularly in regions such as the fusiform gyrus (Andrews et al., 2002) and prefrontal cortex (Michel, 2022). In addition to this, a core aim of the study is to evaluate which ERP component, P100 or N170, is more suitable for capturing the neural differences between conscious and unconscious emotional face processing. This will allow us to identify which electrophysiological marker best differentiates the two levels of perceptual awareness, thus providing a more precise tool for future research in unconscious emotional processing. Specifically, we emphasize that unconscious processing of emotional stimuli is a crucial mechanism to evaluate our everyday surroundings, encode social cues and perform social interactions (Prabhakaran and Gray, 2012; Schütz et al., 2020). Furthermore, individuals suffering from varying mental disorders, such as depression or panic disorder, show alterations in this rapid and unaware processing of social cues such as facial expressions (Baroni et al., 2021; Hahn and Goedderz, 2020; Schräder et al., 2024). Therefore, unconscious behavior/processing can lead to develop and maintain mental disorders (Disner et al., 2011).

Event-related potentials (ERPs) are described as neural responses induced by specific stimuli (Blackwood and Muir, 1990). They allow a time-precise analysis of cognitive processes (Woodman, 2010), which has been considered particularly beneficial in researching emotion processing (Dickey et al., 2021). In this study, different ERP components (P100, N170) were considered as face sensitive early processing potentials.

The positive P100 component peaking at around 100 ms after stimulus onset reflects very early basic visual processing activity. Some research suggests that visual attention has an effect on the P100 (Mangun and Hillyard, 1991; Mangun, 1995). Attended stimuli elicit larger P100 amplitudes than non-attended stimuli (Di Russo and Spinelli, 1999; Hillyard et al., 1998). Other studies report that early face processing is not modulated by attention (Lueschow et al., 2004; Furey et al., 2006; Biehl et al., 2013). The P100 is considered sensitive to faces, with non-facial stimuli evoking smaller P100 amplitudes than facial stimuli (Herrmann et al., 2005a, 2005b; Biehl et al., 2013). Research is not consistent whether P100 amplitudes show variations contingent upon the specific facial emotions presented. For example, one study reports increased P100 amplitudes in response to happy compared to neutral facial expressions (Zhang et al., 2016). Other studies found no significantly different P100 amplitudes between any of the analyzed emotions (sadness, fear, disgust, anger, neutral, surprise, happiness; Batty and Taylor, 2003), or documented an absence of specific emotional effects on the P100 (Smith, 2012). Using different primer presentation times (17 vs. 200 ms), the P100 was additionally found to be sensitive to conscious versus unconscious processing of faces showing higher amplitudes in the conscious state (Zhang et al., 2012). Here, a presentation time of 16.7 ms is considered optimal for unconscious processing (Schräder et al., 2023).

The N170 is an ERP component with a negative peak at around 170 ms post stimulus onset and is considered specific to face stimuli (Bentin et al., 1996; Itier and Taylor, 2004; Hinojosa et al., 2015). Whereas not all studies report significant effects of facial emotion expressions on the N170 (Herrmann et al., 2002; Eimer and Holmes, 2002; Eimer et al., 2003; Ashley et al., 2004; Brunet, 2023), the ERP is found to be modulated by different emotional expressions in several studies (e.g., Batty and Taylor, 2003; Miyoshi et al., 2004; Blau et al., 2007; Hendriks et al., 2007; Zhang et al., 2012, 2016). Variation in referencing methods could explain the differing results observed in previous literature (Rellecke et al., 2013). However, a throughout review summarized evidence for varying N170 amplitudes indicate a heterogeneous sensitivity towards emotional cues (for review see Hinojosa et al., 2015). In healthy controls, joyful or happy faces elicit larger N170 amplitudes than sad faces (Jaworska et al., 2012; Zhang et al., 2016). Enhanced N170 amplitudes have also been found with subliminally presented emotional stimuli (Smith, 2012; Zhang et al., 2012). Furthermore, the N170 is modulated by stimulus presentation time, as stimuli presented for 200 ms elicited larger N170 amplitudes than stimuli presented for 17 ms (Zhang et al., 2012).

A previous study examined the effect of emotion (happy, sad, and neutral) on different ERPs in an established backward masking paradigm (Zhang et al., 2016), yet without comparing conscious versus unconscious processing. We here investigate the effect of the same emotions on the P100 and N170 comparing conscious versus unconscious processing (primary aim). Since supraliminal face stimuli induce larger N170 amplitudes than subliminally presented faces (Zhang et al., 2012; Navajas et al., 2013), we hypothesize that N170 amplitudes will be larger in conscious than in unconscious trials, as conscious perception of faces allows for more detailed and prolonged engagement of cortical regions such as the fusiform face area (FFA), which are critical for fine-grained facial feature analysis (Zhang et al., 2012). In contrast, unconscious presentation, which primarily engages rapid subcortical pathways (e.g., amygdala), may not provide sufficient time for full cortical engagement, resulting in attenuated N170 amplitudes. For the P100, we expected higher amplitudes in unconscious trials, reflecting early attentional mechanisms that may be enhanced due to the brief and automatic nature of unconscious face processing (Zhang et al., 2012). As a secondary aim (additional to pre-registered aims), a sensitivity to emotions is assumed. Trials with happy or sad faces are expected to elicit larger P100 and N170 amplitudes compared to neutral faces.

The target stimulus analysis investigating the effect of emotional conflict on different ERPs (N400, late positive potential (LPP)) can be found in the supplements as exploratory analyses, additional to pre-registered aims.

As face processing is a complex neurological task, several factors may be causal for a modulation of ERPs. In consideration thereof, also brain hemisphere/electrode location, sex and age of the participant will be examined in this study, in addition to presentation time and emotion. Sex differences in face processing were previously reported, e.g., while presenting emotional infants’ faces (Proverbio et al., 2006). This study aims to investigate whether sex differences occur when presenting emotionally expressive adult faces as stimuli. Since previous research suggested right hemisphere dominance during face processing (Bentin et al., 1996; Bötzel et al., 1995; Haxby et al., 1999; Kanwisher et al., 1997; Nguyen and Cunnington, 2014), lateralization with more dominant enhancement of the P100 and N170 amplitudes to the right brain hemisphere is hypothesized. Finally, previous research revealed differences in N170 amplitudes depending on the age of the participants (Taylor et al., 1999; for a review see Taylor et al., 2004).

The backward masking paradigm is often used to prevent awareness and conscious perception of stimuli (Esteves and Ohman, 1993; Liddell et al., 2004; Morris et al., 1998, 1999; Whalen et al., 1998). Utilizing this, previous literature demonstrated the suitability of backward masking specifically for researching unconscious emotion processing by analyzing different ERPs (Balconi and Mazza, 2009). The paradigm allows to investigate differences between conscious and unconscious processing depending on the masking as well as the impact of emotional conflict on emotion processing.

## Materials and methods

The task analyzed here was part of a simultaneous EEG-fMRI study examining unconscious emotional conflict including other tasks and fMRI data which will be published elsewhere. The complete study is preregistered at the open science framework (doi: 10.17605/OSF.IO/37XD2).

### Participants

Healthy participants (*N* = 52; 29 women) aged from 18 to 55 years (M = 26.67, SD = 6.92) were recruited through public advertisements and flyers. All participants were required to be right-handed, have normal or corrected-to-normal vision and fulfill MR-scanning criteria. To ensure that all questionnaires could be answered, fluency in German was required. Participants with acute or history of neurological illnesses, substance-related or psychiatric disorders were excluded.

Due to poor EEG signal quality, one participant had to be excluded from the EEG data analysis reducing the final sample to 51 participants (28 women) with a mean age of 26.73 years (SD = 6.98).

The study protocol was conducted in accordance with the Declaration of Helsinki (World Medical Association, 2013) and was approved by the Ethics committee of the RWTH Aachen Medical Faculty. All participants gave written informed consent and received a financial compensation of 45 Euro.

### Study procedure

Data collection took place over a period of 18 months at the Department of Psychiatry, Psychotherapy, Psychosomatics, Medical Faculty RWTH Aachen University.

Included participants were invited to a 3.5-h measurement appointment. First, participants completed questionnaires. A 64-channel EEG system (BrainAmp MR Amplifier, Brain Products GmbH, Gilching, Germany; BrainAmp [Apparatus], 2023) with an MR-compatible electrode cap was placed on participants’ heads. The 64 electrodes (5 kΩ resistors) included an electrocardiogram electrode (ECG) placed on the back left to the spine, the vertex electrode (Cz) on the middle of the scalp was used as reference electrode.

For the simultaneous EEG-fMRI measurement, participants were positioned in a 3 Tesla MR scanner (Siemens MAGNETOM Prisma®, Siemens Medical Systems, Germany) while their right hand was placed on an MRI compatible button device. For synchronization of EEG and MRI recording a synchronizing box (SyncBox MainUnit, BrainProducts, Germany) was used. To stabilize the head position, a cervical collar was placed.

The measurement session started with performing anatomical brain measurements. After completing two tasks in the scanner participants were asked to fill in a task evaluation questionnaire.

#### Questionnaires

Basic demographic data and past and present medical history of psychiatric disorders were collected using a German version of the Structured Clinical Interview for DSM-5® Disorders (SCID-5; Beesdo-Baum et al., 2019). The Trail Making Test (TMT; Reitan, 1971) was used to evaluate visual processing speed, working memory and executive functions (Tischler and Petermann, 2010). To assess intelligence, a multiple-choice word test (Mehrfachwahl-Wortschatz-Intelligenztest, MWT-B) was used (Lehrl, 1977). Intelligence level was determined by age and sex matched norms. Short term memory was tested using a subtest (digit span) of the Wechsler Memory Scale (WMS-R; Härting and Wechsler, 2000). The Bermond-Vorst Alexithymia Questionnaire (BVAQ; Vorst and Bermond, 2001) was applied to test for possible occurrence of alexithymia symptoms. To evaluate individual perception of the task, participants received an additional questionnaire after completing the tasks. This questionnaire assessed the subjective difficulty of evaluating emotion (1 = *easy*; 2 = *rather easy*; 3 = *moderate*; 4 = *rather difficult*; 5 = *difficult*). Due to missing data, two participants had to be excluded from the statistical analysis of this questionnaire.

#### Modified backward masked emotional conflict task

In the modified backward masked emotional conflict task, images of facial expressions showing three different types of emotion (happy, sad, or neutral) were presented in line with previous research (Zhang et al., 2016; Stuhrmann et al., 2013; Chen et al., 2014; Xu et al., 2013). In total, 36 faces (12 faces for each of the three emotions) with an equal number of men/women per emotion were taken from the FACES database (Ebner et al., 2010). A scrambled checkerboard pattern mask was built by Adobe Photoshop® based on a picture selected from the FACES database to ensure equal luminance between mask und stimuli. Images were shown against grey background at the center of an LCD monitor (120 Hz refresh rate) using PsychoPy3 software (Peirce, 2007).

Each trial started with a white fixation cross for a duration of 300 ms (36 frames) which was followed by a primer image. Since presentation time of 16.7 ms was found to be ideal for unconsciously perceived stimuli (Schräder et al., 2023), unconscious primers were shown for 16.7 ms (2 frames) while conscious primers were presented for 150 ms (18 frames). Prime stimuli were followed by the mask which was displayed for 66.7 ms (8 frames). The mask was followed by a target image presented for 300 ms.

**Figure 1:**
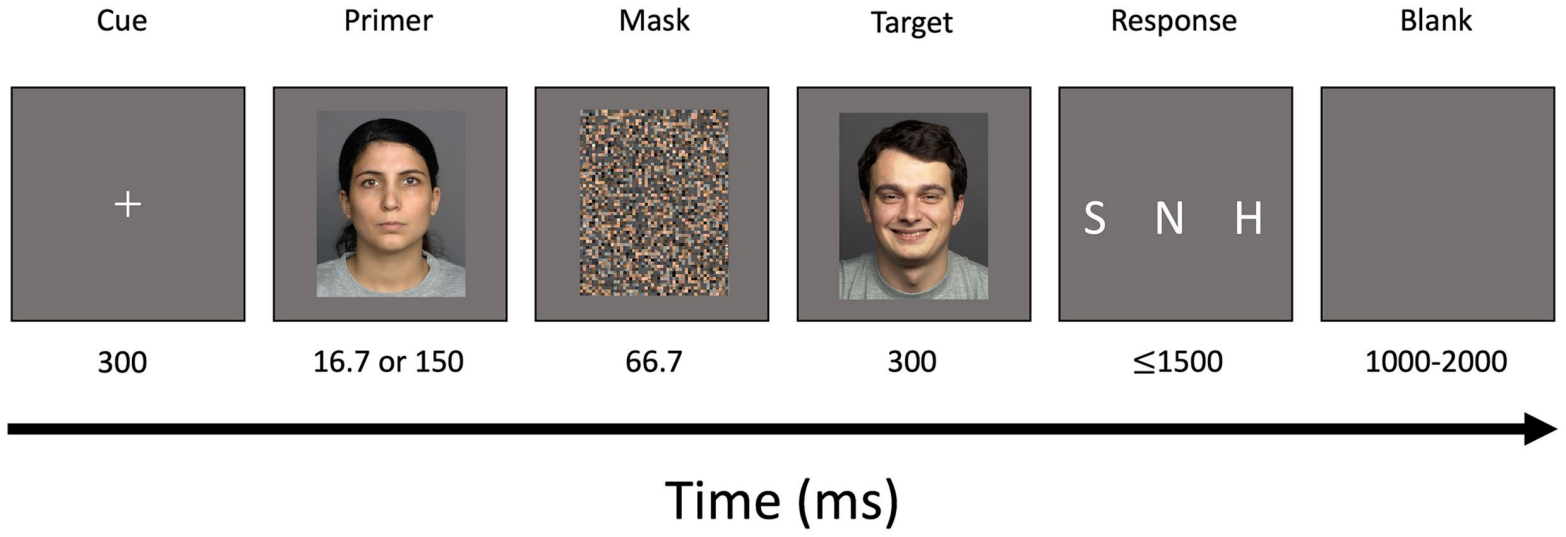
Schematic illustration of modified backward masked emotional conflict task (incongruent trial). A centered white fixation cross was displayed for a duration of 300 ms. Primers depicting happy, sad, or neutral facial expressions were presented for a duration of either 16.7 ms (unconscious trials) or 150 ms (conscious trials). A scrambled mask was shown for 66.7 ms followed by a target image also depicting happy, sad, or neutral facial expressions. A response screen was presented for 1,500 ms followed by a blank screen for a duration of 1,000–2,000 ms. S, sad; N, neutral; H, happy.

Afterwards, a response screen appeared for 1.5 s. Participants were asked to decide if the facial expression of the target image was “happy,” “sad,” or “neutral” as fast and as accurately as possible using three response buttons at the right hand. The index finger was used to indicate “sad,” middle finger for “neutral” and ring finger for “happy” faces. The response screen was followed by a blank screen serving as a jitter for a random duration between 1 to 2 s (Figure 1).

The task consisted of 3 blocks of each 120 trials in pseudorandomized order with a matching number of emotion-congruent and incongruent prime-target pairs. Each face served a maximum of 12 times as a target stimulus. Two practice trials were performed at the beginning.

The experimental set-up created a 2 × 2 × 3 factorial design (unconscious vs. conscious primer; primer-target emotion congruent vs. incongruent; emotion happy vs. sad vs. neutral) resulting in 30 trials for each of the 12 conditions.

The task lasted about 30 min.

### EEG data preprocessing

EEG data were recorded with the BrainVision Recorder software (BrainVision Recorder, Vers. 1.23.0003, Brain Products GmbH, Gilching, Germany; BrainVision Recorder (Vers. 1.23.0003) [Software], 2023) at a 1,000 Hz sampling rate (0.01–250 Hz analog band-pass filter). The BrainVision Analyzer software (BrainVision Analyzer, Version 2.2.0, Brain Products GmbH, Gilching, Germany; BrainVision Analyzer (Version 2.2.0) [Software], 2019) was used for EEG data preprocessing. The simple gradient method was used to remove MR scanner artifacts (Allen et al., 2000). An infinite impulse response filter (IIR, 70 Hz cut off, 48 dB slope) was applied and data were down-sampled to 500 Hz which acts as a low-pass-filter. Data then underwent cardioballistic pulse artifacts detection and correction (Allen et al., 1998). Linear topographic interpolation was performed on EEG channels selected after visual inspection. After down-sampling to 250 Hz, data were filtered (0.01–45 Hz, 24 dB slope; ECG channel excluded from filter). Independent component analysis (ICA) was used to identify, e.g., eye blink artifacts which were removed from the data. All datasets were visually inspected, and intervals of bad quality were semi automatically detected and rejected. EEG data were exported for further analyses via EEGLAB (MATLAB®).

### EEG data processing

Further EEG data processing was done by using MATLAB software (version 9.9.0.1467703 (R2020b). Natick, Massachusetts: The MathWorks Inc, 2020) and the implemented toolbox EEGLab (version 2022.1; Delorme and Makeig, 2004). The preprocessed EEG data were segmented to epochs ranging from −200 to 800 ms relative to the primer onset (Nguyen and Cunnington, 2014). For target analysis, epochs were expanded to a range from −200 to 1,200 ms relative to primer onset. Baseline correction for the interval of 200 ms prior to the primer onset was carried out (Nguyen and Cunnington, 2014; Zhang et al., 2016).

**Figure 2:**
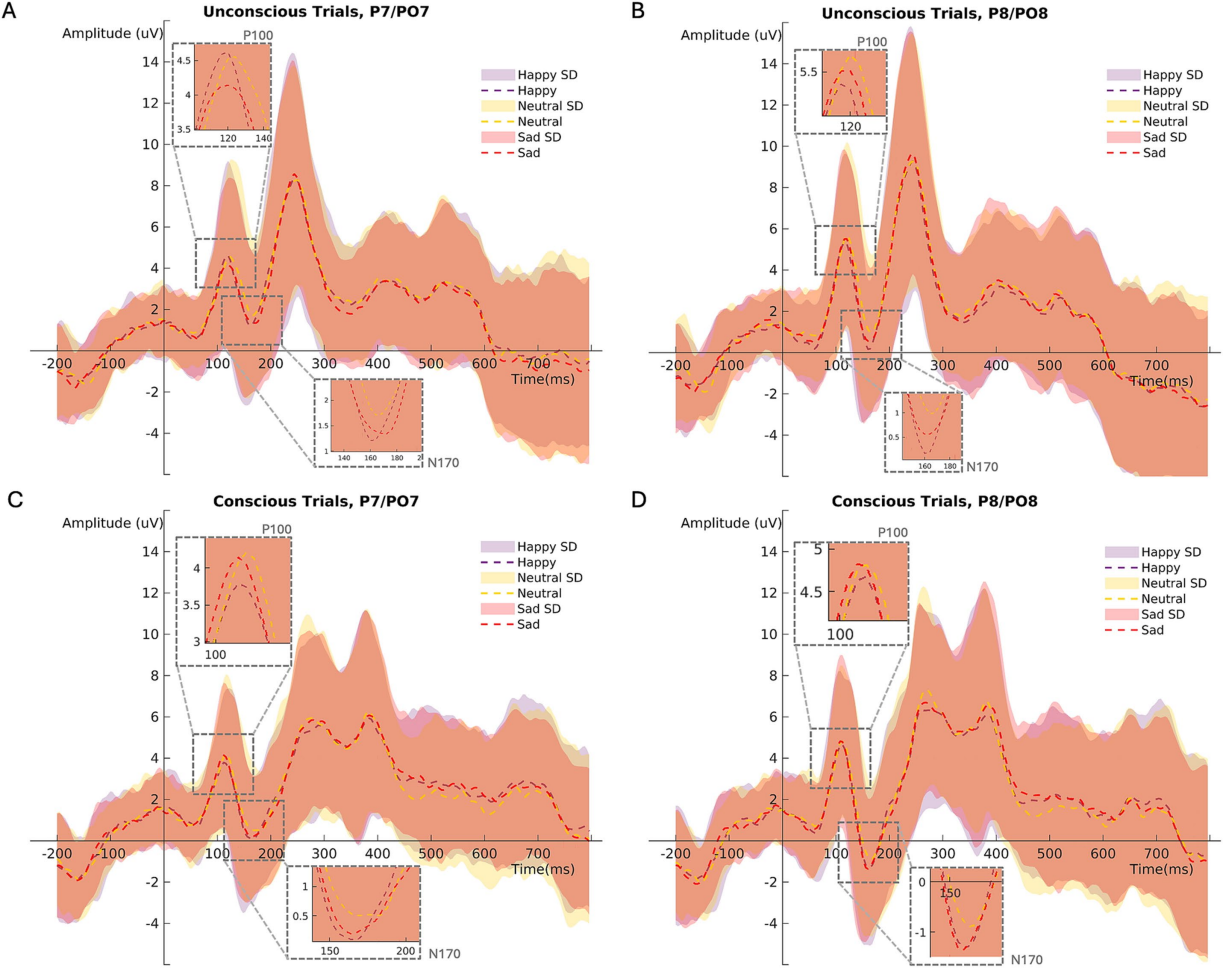
Mean ERP (P100/N170) amplitudes at channels PO7/P7 (left hemisphere) during **(A)** unconscious, **(C)** conscious trials. Mean ERP amplitudes at channels PO8/P8 (right hemisphere) during **(B)** unconscious, **(D)** conscious trials. SD = standard deviation.

For ERPs analysis referring to the primer (P100 and N170), the channels P7, PO7, P8 and PO8 were chosen (Nguyen and Cunnington, 2014). Mean ERPs of the channels P7 and PO7 (left hemisphere) as well as the channels P8 and PO8 (right hemisphere) were created for every participant. For the primer analysis, P100 and N170 were computed for every subject by averaging the segments for each primer condition (happy, sad, and neutral primer emotion × unconscious and conscious primer presentation; Figure 2).

The N170 peak component (negative) was estimated between 152 to 200 ms after primer stimulus onset (Hinojosa et al., 2015). Following previous studies, the P100 time interval was initially set to 80 to 120 ms (Nguyen and Cunnington, 2014). After visual data inspection, the P100 peak component (positive) was estimated in an extended time interval ranging from 80 to 132 ms after primer stimulus onset.

The identified negative (N170) and positive (P100) peaks within the time windows were subjected to statistical analysis using R (R Core Team, 2022) and RStudio (version 2022.12.0.353; Posit team, 2022).

### Statistical analysis

#### Questionnaires

The data obtained through the questionnaires were analyzed using IBM SPSS statistics [version 28.0.0.0 (190)]. To compare participants regarding sex differences, independent samples t-tests were conducted at a significance level of α = 0.05. Spearman’s Rho was computed to assess the relationship between the N170 and subjective difficulty of evaluating emotions in the paradigm. An analysis of possible relationships between different questionnaires and the ERP components can be found in the supplements (Supplementary Tables S1, S2).

#### Event-related potentials

Linear mixed models (LMMs) using the lme4 package (Bates et al., 2015) from R^1^ were used to conduct EEG data analysis. Using LMMs allows accounting for individual differences among participants as well as correlations between repeated measurements within individuals. Employing LMMs instead of traditional ANOVA significantly increases the degrees of freedom in the analysis enhancing the power and sensitivity to detect small main effects, making our design more robust than what would be achieved with ANOVA (Matuschek et al., 2017; de Melo et al., 2022).

For primer analysis, P100 (Model1) and N170 (Model2) served as dependent variables in their respective models. Set as fixed effects were presentation time (mask; conscious or unconscious), emotion (primer happy, sad, or neutral), sex (male or female), hemisphere (left or right) and age. A random intercept for each participant was included.

Model1 <- lmer(P100 ~ mask + primer emotion + sex + hemisphere + age+ (1|participant))

Model2 <- lmer(N170 ~ mask + primer emotion + sex + hemisphere + age + (1|participant))

During the model selection process, linear mixed models without interaction between variables were chosen due to better model quality criteria (AIC/BIC; see Supplementary material). Effect sizes were calculated using squaredGLMM and powerSim function in R.

#### Accuracy

Using the MASS package (Venables and Ripley, 2002) from R (https://cran.r-project.org/), a general linear mixed model (GLMM) was computed applying the Penalized Quasi-Likelihood.

For analysis of participants’ accuracy (Model5) in evaluating the target emotion, the accuracy served as the dependent variable. Target emotion (happy, sad, or neutral), primer presentation time (consciousness; conscious or unconscious perception), congruency (primer-target emotion congruent or incongruent), age and sex were included as fixed effects. A random intercept was included for each participant. Using the emmeans package (Lenth, 2023) estimated marginal means were obtained and subsequently used for pairwise comparisons as *post hoc* tests.

Model5 <- glmmPQL(accuracy ~ target emotion + consciousness + congruency + age + sex, random = ~ 1|participant, family = quasipoisson(link = “log”))

## Results

### Questionnaires

No statistically significant differences regarding age, and questionnaire scores were found between men and women (Table 1).

**Table 1:** Descriptive statistics of questionnaire scores and age.

Questionnaire	Men (*n* = 23)	Women (*n* = 29)					
	*M*	*SD*	*M*	*SD*	*t*	*df*	*p*
BVAQ (Sum)	49.96	7.13	47.86	7.12	1.05	50	0.297
MWT-B (Norm)	0.52	0.84	0.42	0.73	0.46	50	0.648
TMT (B/A Norm)	0.13	1.11	−0.05	1.62	0.46	50	0.648
WMS-R Forward (Sum)	11.61	2.17	11.59	2.72	0.03	50	0.974
WMS-R Backward (Sum)	8.91	2.35	7.59	2.71	1.86	50	0.069
Age	25.91	5.59	27.28	7.97	−0.70	50	0.490

BVAQ, Bermond-Vorst Alexithymia Questionnaire; MWT-B, Mehrfachwahl-Wortschatz-Intelligenztest (engl. multiple choice verbal intelligence test); TMT, Trail Making Test; WMS-R, Wechsler Memory Scale-Revised. “sum”, total score, “norm”, normalized score.

For an exploratory analysis of effects of different questionnaire scores on the ERP components as well as participants’ accuracy, see Supplementary material.

### Event-related potentials

#### P100

The linear mixed model (LMM) testing task effects on the P100 revealed a significant effect of mask (*F*(1,557) = 14.78, *p* < 0.001, partial Eta^2^ = 0.03, CI [0.01, 1.00]), sex (*F*(1,48) = 4.08, *p* = 0.049, partial Eta^2^ = 0.08, CI [0.00, 1.00]) and brain hemisphere (*F*(1,557) = 40.64, *p* < 0.001, partial Eta^2^ = 0.07, CI [0.04, 1.00]). No significant effect was found for primer emotion and age (Table 2).

**Table 2:** Effect on P100 values: Type II analysis of variance table with Kenward-Roger’s method for P100 model.

Measure	Sum Sq	Mean Sq	NumDF	*df*	*F* value	*p* value	Power	Cohen’s f	Partial Eta2	CI
Mask	56.07	56.07	1	557	14.78	<0.001^***^	96.9%	0.0058	0.03	[0.01, 1.00]
Primer emotion	0.02	0.01	2	557	0.003	0.997	5.3%	−0.0001	<0.001	[0.00, 1.00]
Sex	15.48	15.48	1	48	4.08	0.049^*^	53.2%	0.0621	0.08	[0.00, 1.00]
Hemisphere	154.14	154.14	1	557	40.64	<0.001 ^***^	99.9%	0.0159	0.07	[0.04, 1.00]
Age	0.51	0.51	1	48	0.13	0.717	6.6%	0.0007	<0.001	[0.00, 1.00]

***p < 0.001.

**Figure 3:**
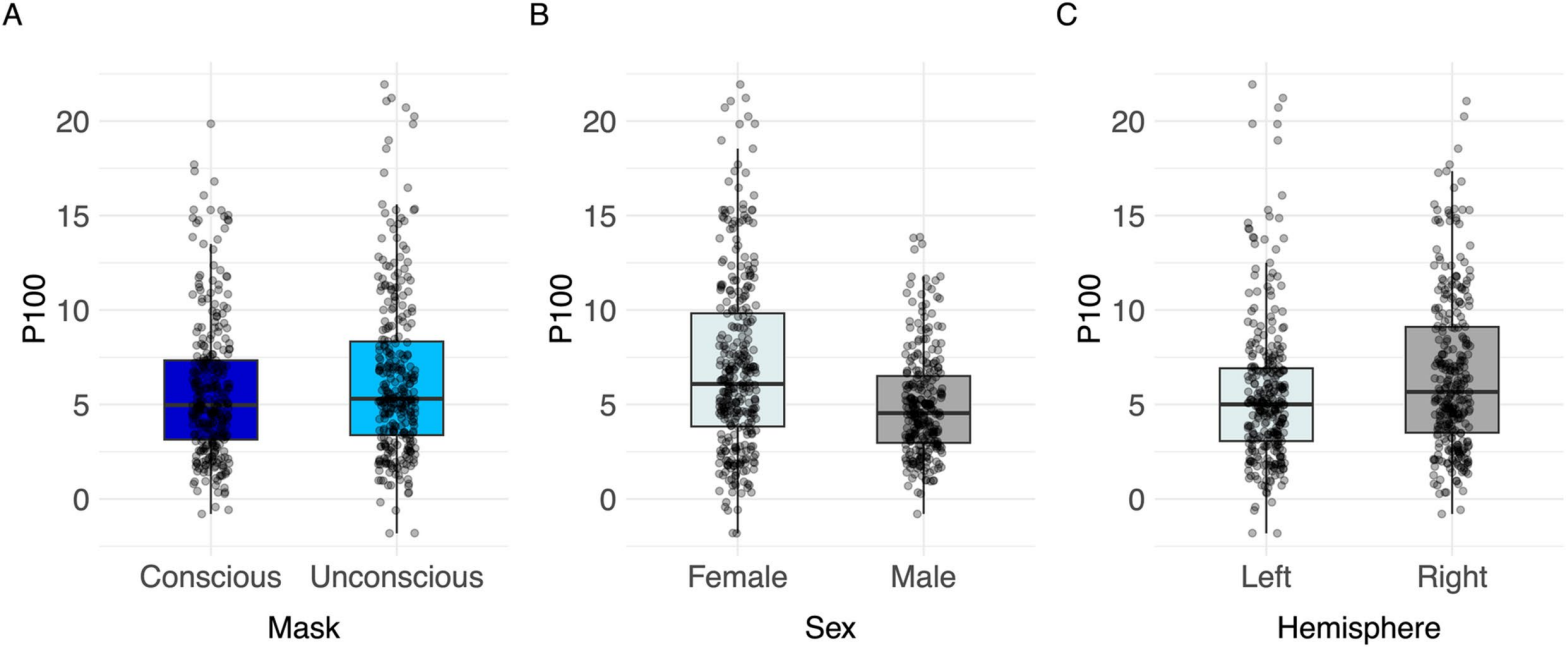
Visualization of parameter effects for P100. Boxplot of **(A)** mask, **(B)** sex, **(C)** brain hemisphere side.

**Figure 4:**
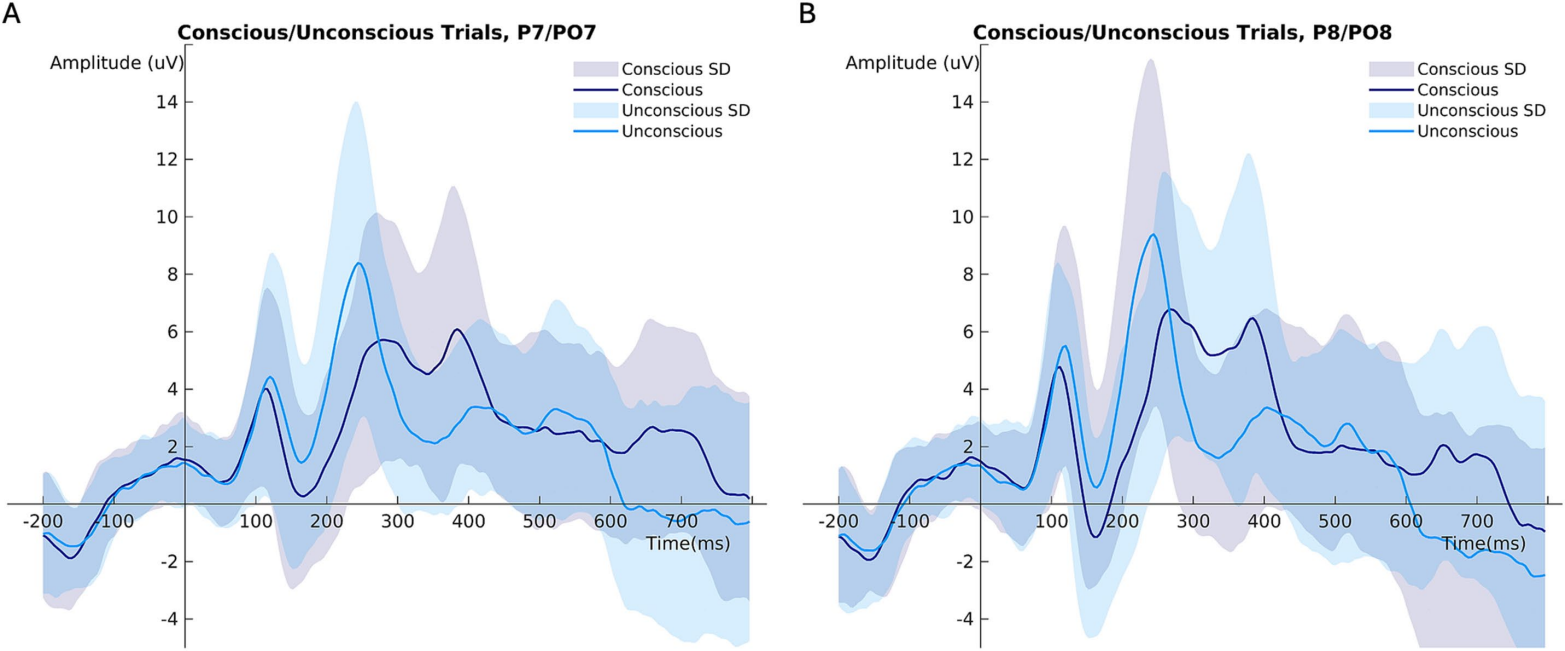
Mean ERP amplitudes comparing conscious and unconscious trials at **(A)** channels P7/PO7 (left hemisphere), **(B)** channels P8/PO8 (right hemisphere). ***p < 0.001.

**Figure 5:**
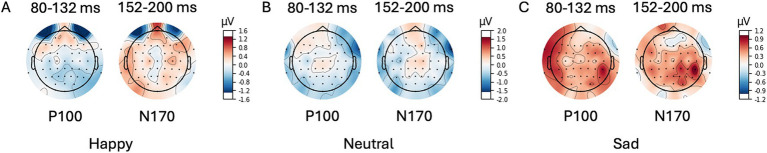
Scalp Topography Maps for P100/N170. Difference (unconscious voltage minus conscious voltage) between average amplitude in time intervals (80–132 ms post primer for P100; 152–200 ms post primer for N170). Primer emotion **(A)** happy, **(B)** neutral, and **(C)** (sad).

In unconscious trials, the P100 amplitude was larger than in conscious trials (Figure 3A; Table 2). The right brain hemisphere (channels P8/PO8) showed a significantly larger P100 in comparison to the left hemisphere (channels P7/PO7) (Figures 3C, 4, 5). Women had larger P100 values than men (Figure 3B; Table 2). The model explained 8.21% of variance via the fixed factors and 77.89% via the random intercept.

#### N170

Significant effects of mask (*F*(1,557) = 79.34, *p* < 0.001, partial Eta^2^ = 0.12, CI [0.09, 1.00]), primer emotion (*F*(2,557) = 3.28, *p* = 0.038, partial Eta^2^ = 0.01, CI [0.00, 1.00])), hemisphere (*F*(1,557) = 50.67, *p* < 0.001, partial Eta^2^ = 0.08, CI [0.05, 1.00]), sex (*F*(1,48) = 4.17, *p* = 0.047, partial Eta^2^ = 0.08, CI [0.00, 1.00])) and age (*F*(1,48) = 4.46, *p* = 0.040, partial Eta^2^ = 0.08, CI [0.00, 1.00]) were found (Table 3).

**Table 3:** Effect on N170 value: Type II analysis of variance table with Kenward-Roger’s method for N170 model.

Measure	Sum Sq	Mean Sq	NumDF	df	F value	*p* value	Power	Cohen’s f	Partial Eta2	CI
Mask	292.27	292.27	1	557	79.34	<0.001^***^	99.9%	0.0436	0.12	[0.09, 1.00]
Primer emotion	24.16	12.08	2	557	3.28	0.038^*^	59.8%	0.0034	0.01	[0.00, 1.00]
Sex	15.38	15.38	1	48	4.17	0.047^*^	52.6%	0.0554	0.08	[0.00, 1.00]
Hemisphere	186.63	186.63	1	557	50.67	<0.001^***^	99.9%	0.0278	0.08	[0.05, 1.00]
Age	16.42	16.42	1	48	4.46	0.040^*^	51.5%	0.0593	0.08	[0.00, 1.00]

*p < 0.05; ***p < 0.001.

**Figure 6:**
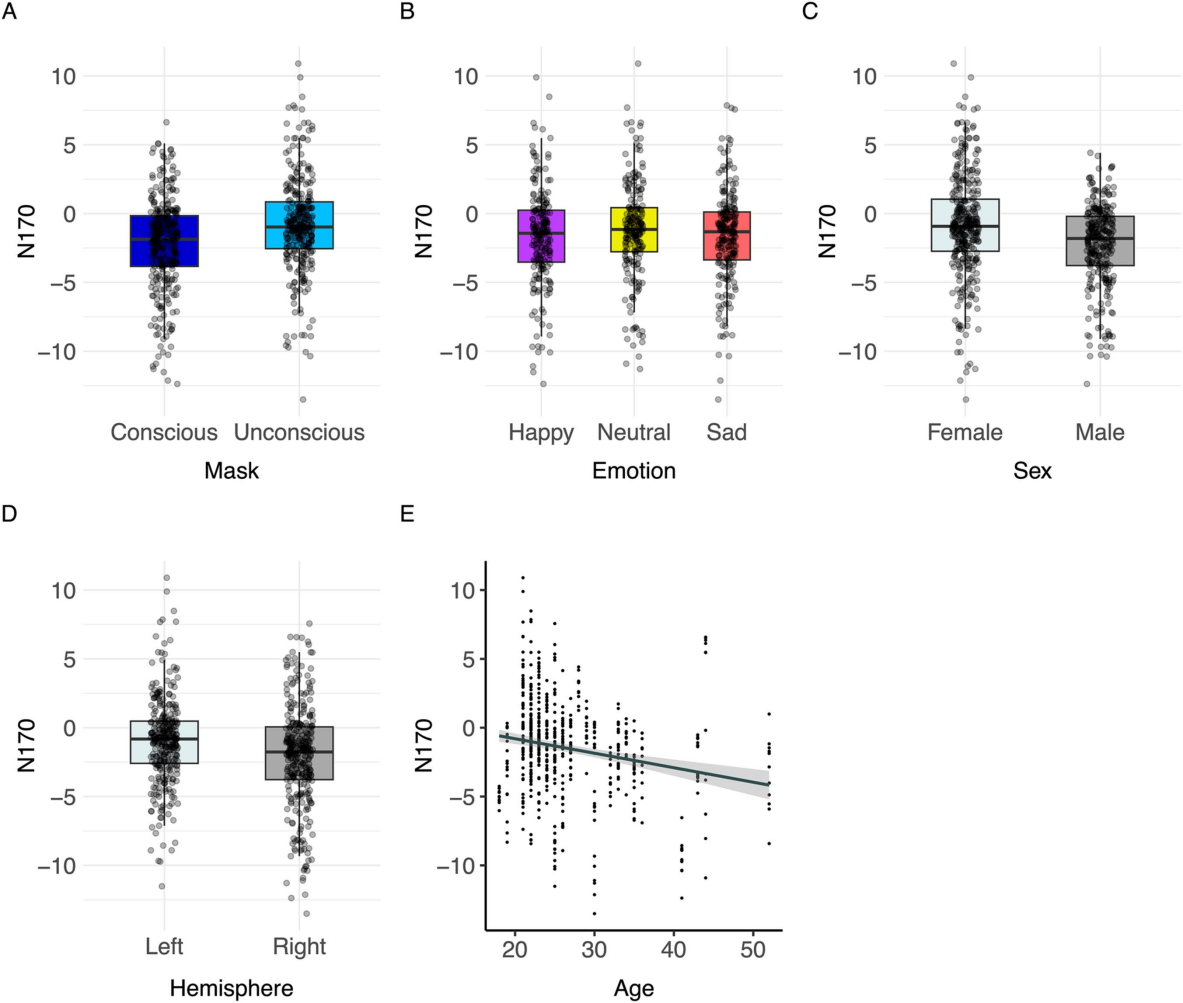
Visualization of parameter effects for N170. Boxplot of **(A)** mask, **(B)** emotion, **(C)** sex, **(D)** brain hemisphere, and **(E)** age.

Conscious trials elicited a significantly larger N170 than unconscious trials (Figure 6A). Men showed higher N170 amplitudes than women (Figure 6C; Table 3). Right hemisphere induced higher N170 amplitudes compared to left hemisphere (Figures 2, 6D, Table 3). *Post hoc* comparisons showed no significant difference between emotions (Figure 6B; Table 4). The model explained 15.34% of variance via the fixed factors and 71.54% via the random intercept.

**Table 4:** Pairwise Contrasts of primer emotion effect for N170 linear mixed model.

Contrast	Estimate	*SE*	*df*	*t* ratio	*p* value
Happy–neutral	−0.43	0.19	557	−2.26	0.063^a^
Happy–sad	−0.01	0.19	557	−0.08	0.997^a^
Neutral–sad	0.41	0.19	557	2.18	0.076^a^

Degrees-of-freedom method: Kenward-Roger.

a *p value adjustment: Tukey method for comparing a family of 3 estimates.*

Spearman’s correlation between N170 values and subjective difficulty of evaluating the target emotions shows a significant positive correlation [Spearman’s *ρ*(588) = 0.242, *p* < 0.001].

### Accuracy

**Figure 7:**
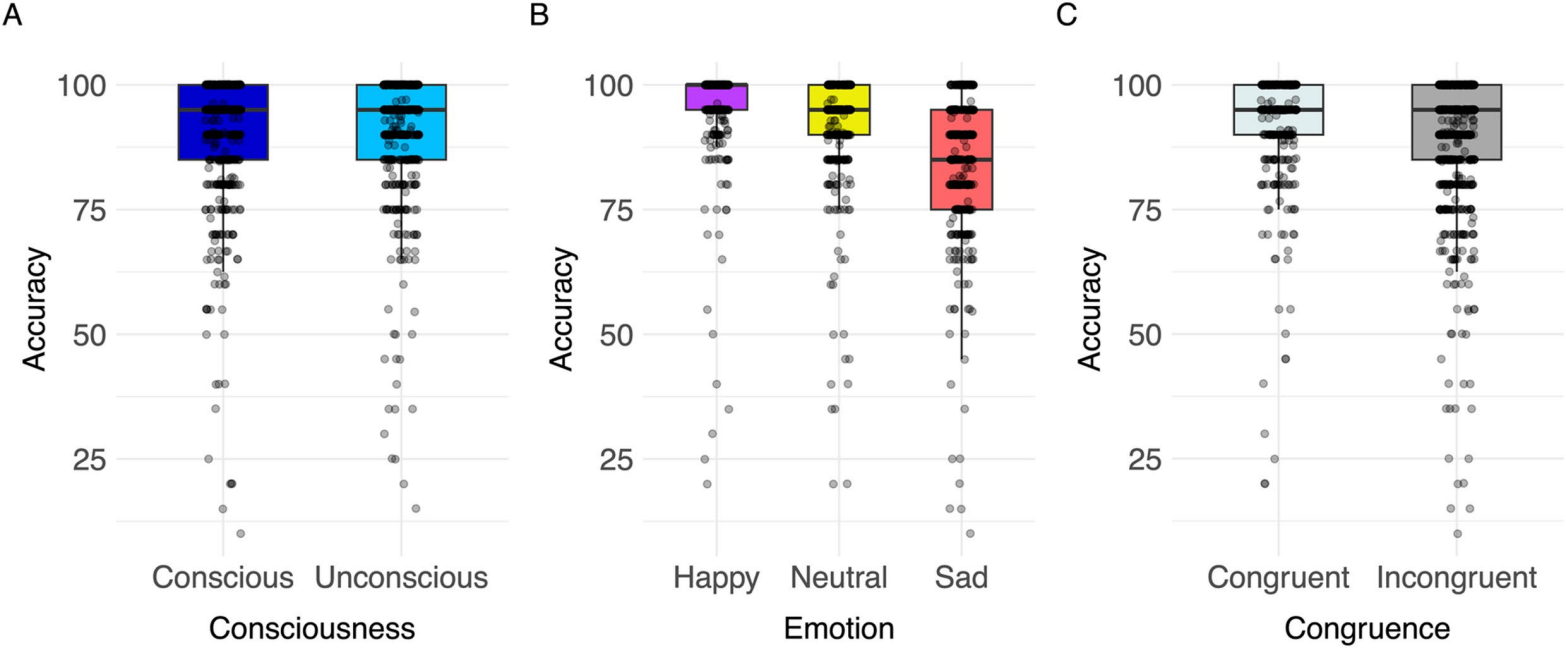
Visualization of parameter effects for accuracy. Boxplot of **(A)** congruence, **(B)** emotion, **(C)** consciousness.

A significant effect of congruence was found [*F*(1,863) = 4.74, *p* < 0.001, Cohen’s *f* = 1.93; for all results see Table 5]. Response accuracy was greater in congruent than in incongruent trials (Figure 7). Post hoc comparisons indicated significant differences between all three target emotions [happy-neutral *t*(863) = 6.31, *p* < 0.001; happy-sad *t*(863) = 17.92, *p* < 0.001; neutral-sad *t*(863) = 11.63, *p* < 0.001; Table 5]. Participants’ responses were most accurate in happy target faces (happy > neutral > sad; Figure 7). The model explained 10.47% of variance via the fixed factors and 79.46% via the random intercept.

**Table 5:** Effect on accuracy.

Measure	Value	*SE*	*df*	*Cohen’s f*	*t* value	*p* value
Congruence	0.02	< 0.01	863	1.93	4.74	<0.001 ***
Emotion						
Happy–neutral	0.05	0.01	863	2.3	6.31	<0.001 ^a^
Happy–sad	0.14	0.01	863	4.12	17.92	<0.001 ^a^
Neutral–sad	0.09	0.01	863	3.26	11.63	<0.001 ^a^
Consciousness	<−0.01	<0.01	863	0.72	−1.52	0.128
Sex	<−0.01	0.02	48	<0.001	−0.19	0.848
Age	<0.01	<0.01	48	0.54	1.29	0.203

***p < 0.001.*p*-value adjustment: Tukey method for comparing a family of 3 estimates.

a Pairwise comparison of target emotion effect on accuracy.

## Discussion

The current study investigated the influence of conscious versus unconscious emotional face processing on electrophysiological markers. The P100 and N170 components were selected to analyze differences in conscious and unconscious emotional face processing. We observed higher accuracy in happy target trials compared to neutral and sad. Stimulus presentation time influenced the P100 and N170 amplitude. While P100 amplitudes were larger in trials with unconscious compared to conscious primer presentation, the N170 showed opposite results. N170 was modulated by emotion with *post hoc* effects being not significant. The N170 was lower when participants reported higher subjective difficulty of evaluating emotions.

### Conscious vs. unconscious processing

The result of larger P100 amplitudes in unconscious than conscious trials is in line with past research. For example, larger P100 amplitudes were reported in trials where facial stimuli were presented for 17 ms (unconscious) in comparison to 200 ms (conscious) presentation time (Zhang et al., 2012). Assuming a modulation of P100 amplitudes by attention as reported in some literature showing higher P100 in trials with increased attention (Mangun and Hillyard, 1991; Mangun, 1995), briefly presented stimuli may elicit higher attention than trials with longer presentation times. This may lead to higher P100 amplitudes due to the increased attentional demand (Hillyard et al., 1998). A review proposed that some unconsciously perceived emotional facial expressions may attract higher attention, aiming to gain awareness of the emotional face (Eastwood and Smilek, 2005). Therefore, attention effects may be highly relevant especially for shortly presented facial expressions and are reflected by P100. Additionally, unconsciously perceived stimuli may have been processed only partly at this early stage which could increase the attentional demand resulting in higher P100 values.

N170 was larger in conscious compared to unconscious trials. The N170 is sensitive to face stimuli (Hinojosa et al., 2015) and fixation on specific face parts, e.g., on eyes elicited larger N170 amplitudes than fixation on mouths (de Lissa et al., 2014). Therefore, trials with longer stimuli presentation times may enable more detailed face processing resulting in larger N170. Our findings are in line with previous research proposing sensitivity of the N170 component to the visibility of faces (Zhang et al., 2012). Our results demonstrate a clear modulation of P100 and N170 amplitudes by stimulus presentation time, reflecting differential neural processing in conscious versus unconscious perception. The larger P100 amplitudes in unconscious trials suggest an early, rapid attentional mechanism that is activated by brief, unconsciously perceived stimuli. This heightened P100 response may indicate that the visual system allocates more attentional resources to quickly process stimuli that are only partially perceived, aligning with the hypothesis that unconscious emotional stimuli can attract higher attention (Eastwood and Smilek, 2005). On the other hand, the N170 amplitude was larger in conscious trials, consistent with the notion that conscious face perception engages more detailed and prolonged visual analysis, allowing for full engagement of face-specific cortical regions such as the fusiform gyrus (Zhang et al., 2012). This ERP has found to be altered depression on an unconscious level as well (Zhang et al., 2016), showing implications on how this ERP can be used to further understand the mechanisms of mental disorders. Furthermore, N170 and P100 can be used to classify unconscious vs. conscious processing and could be used to study more real life-like situations including subliminal facial expression processing using, e.g., dynamic stimulus material and can be therefore adapted to elaborate on disease specific alterations in unconscious emotion processing. Unconscious processing has practical relevance in daily interactions, where subtle emotional cues in faces may be registered unconsciously, influencing reactions before conscious awareness. This type of rapid processing could assist in social situations by helping to assess emotions or detect threats without requiring full awareness. Neuroscientific evidence supports the biological plausibility of processing complex stimuli like faces without awareness. Studies indicate that brain regions like the amygdala and subcortical pathways respond to faces even when not consciously perceived, suggesting an evolutionary adaptation for rapid, unconscious assessment of socially relevant cues (Pessoa, 2005; Tamietto and de Gelder, 2010). These findings align with our study’s aim to explore unconscious processing, showing its importance beyond controlled settings.

### Emotion effect on ERP signals

We hypothesized that the P100 component might be larger in trials displaying happy or sad faces compared to trials with neutral faces. However, the P100 was not significantly affected by the presented emotion in our study which could be due to a small effect size or the power of our model including the factor “emotion.” Since we observed wide confidence intervals that indicate a large data variability, we assume different sources that are not captured in our model. With a larger sample size, we could enhance precision about the effect and report more reliably reproducible effects. Other studies did not find an emotion effect on P100, as well (Batty and Taylor, 2003; Smith, 2012; Lee et al., 2017). The deviating results may be explained by the use of different methods calculating the ERP amplitudes, averaging the amplitude in a given time window (Zhang et al., 2016), contrary to selecting the peak within the time window. Another possible explanation may be differences regarding the experimental design, e.g., the longer stimulus presentation time of 500 ms, or the absence of a mask (Moradi et al., 2017).

As indicated by previous results (Hinojosa et al., 2015; Batty and Taylor, 2003; Miyoshi et al., 2004; Blau et al., 2007; Hendriks et al., 2007; Pegna et al., 2011; Smith, 2012; Zhang et al., 2012, 2016), the N170 component was influenced by different emotions. While the main effect of emotion on N170 was significant, *post hoc* comparisons between primer emotions were not significant. Since confidence intervals were wide and included the null value, large data variability might be considered as an influencing factor on the results. A meta-analysis underlines the effect of the emotion ‘happy’ on the N170 amplitude compared to neutral faces, whereas it does not substantiate the effect of the emotion ‘sad’ compared to neutral faces (Hinojosa et al., 2015).

Despite the robust effect of happy emotions, there are also contradictory findings with larger N170 amplitudes in sad faces than in happy or neutral faces (Lynn and Salisbury, 2008). There might be interindividual differences and preferences towards a specific emotion facilitating its processing. Assuming that such a bias towards specific emotions exists, this would result in increased ERPs for a specific emotion. In our study, the results would not support a specific bias towards one specific emotion, as no significant difference comparing happy and sad emotional faces was found. However, others found biases towards emotional information (for review see Kauschke et al., 2019).

We found a positive correlation between the N170 and subjective difficulty of evaluating emotion within the task paradigm. Participants who reported emotion assessment to be easier, elicited larger N170 amplitudes. Therefore, the emotion effect on the N170 may be dependent on participants’ general sensitivity and ability to discriminate emotions.

Early ERPs were found to be associated with lower-level processing, whereas later ERPs reflected more complex cognitive processes (Sur and Sinha, 2009; Portella et al., 2012). Even though the temporal distance between P100 and N170 is short, this is a substantial duration in the context of brain processing. This trend may be reflected by the finding that the N170 amplitude was modulated by presentation time as well as emotion, while the earlier P100 component was only influenced by stimulus presentation time.

### Other potential influencing factors on ERP components

The present study found significantly larger P100 amplitudes in women compared to men, whereas the N170 showed opposite results. Previous work investigating the effect of sex on ERPs related to emotional face processing found that women elicited larger P100 amplitudes than men in subthreshold fearful faces (Lee et al., 2017). Using an oddball paradigm, one study found that women elicited larger N170 amplitudes in emotional compared to neutral faces, whereas no such effect was found in men (Choi et al., 2015). Since previous study designs differed in experimental paradigm and emotions presented, it is unclear whether these findings are applicable to discuss our results. As the amount of research conducted on the effect of sex on the P100 and the N170 component is still insufficient, further research is needed for conclusive results. Additionally, it should be considered that the confidence intervals were wide indicating larger data variability and included the null value highlighting the need for more extensive research on the influence of the factor “sex” on the ERP components.

Both the P100 and the N170 amplitudes were larger on the right compared to the left hemisphere. In accordance with this, past findings suggest right hemisphere dominance for face processing in the N170, whereas results on the P100 are not fully conclusive yet (e.g., Leehey et al., 1978; Levine et al., 1988; Bötzel et al., 1995; Bentin et al., 1996; Kanwisher et al., 1997; Haxby et al., 1999; Rossion et al., 2003; Itier and Taylor, 2004; Zhang et al., 2012; Nguyen and Cunnington, 2014).

The N170 was increased with higher age which is in line with previous studies which discovered a trend towards an increase in N170 amplitudes in older participants. However, these studies were mainly focused on the population of children and adolescents (e.g., Taylor et al., 1999; for a review see Taylor et al., 2004). One study reported no significant difference of N170 amplitudes between young and middle-aged participants (Shi et al., 2022). Considering the face sensitivity of the N170 (Bentin et al., 1996; Hinojosa et al., 2015), it could be hypothesized that face processing still develops and matures in adults over the course of life. Accounting for the width and inclusion of the null effect in the confidence intervals analyzing the effect of age on the N170, our results should only be applied cautionary to other populations and more research on the effect of age in adult populations on the amplitude of the P100 and the N170 is required to establish conclusive results.

## Limitations

To avoid introducing noise into the ERP data caused by possible exhibition of atypical P100 and N170 responses in neurodivergent participants, only healthy participants were included. However, investigating a larger and more diverse sample could make the results more reliable.

Emotions in the paradigm were limited to happy, sad, and neutral facial expressions. Using a wider variety, e.g., additionally angry, and fearful faces could broaden the results. Additionally, our experiment is limited in investigating the actual perceived awareness of a stimulus in a trial but can only refer to mean ratings retrospectively.

## Conclusion, implications, and outlook

Our results revealed that the P100 and the N170 components were modulated by conscious versus unconscious presentation times. While briefly presented faces elicited larger P100 amplitudes, N170 amplitudes showed opposite results. Consequently, P100 and N170 may be used as sensitive electrophysiological markers for investigating differences between conscious and unconscious emotional face processing. The N170 component was found to be enhanced in response to happy and sad facial expressions compared to neutral, which suggests a non-specific emotion effect.

## Data storage

All acquired data are electronically stored in a pseudonymous form for a duration of 10 years on the server and external hard drives in the Brain Imaging core facility of the IZKF Aachen (Interdisciplinary Centre for Clinical Research).

## Data Availability

The datasets presented in this study can be found in online repositories. The names of the repository/repositories and accession number(s) can be found at: https://github.com/LennardHerzberg/MarkersUnconsciousProcessing.

## Footnotes

^1^ https://cran.r-project.org/
