# Supplementary material for: Identifying P100 and N170 as electrophysiological markers for conscious and unconscious processing of emotional facial expressions
Source: Front Behav Neurosci. 2025 Jan 23;18:1464888. doi: 10.3389/fnbeh.2024.1464888 (PMC11798883; doi:10.3389/fnbeh.2024.1464888)
Supplement: Supplementary file 1 [file Data_Sheet_1.docx]

***Supplementary Material***

**Introduction**

In an exploratory analysis, the N400 and the late positive potential (LPP) were tested for their sensitivity to early incongruency processing in the context of emotional facial expressions.

The N400 is a negative ERP component peaking around 400 ms post stimulus (Kutas & Hillyard, 1980) and is frequently analyzed in the context of semantic incongruencies (for review see Kutas & Federmeier, 2011). The majority of previous research reports larger N400 amplitudes in incongruent compared to congruent trials (e.g. Eder et al., 2012; Morris et al., 2003; Steinbeis & Koelsch, 2011; Zhang et al., 2006; Yu et al., 2022; Schauenburg et al., 2019; for review see Kutas & Federmeier, 2011), but there are also opposite results (Hietanen & Astikainen, 2013; Paulmann & Pell, 2010) or studies stating no significant differences (Hinojosa et al., 2009; Herring et al., 2011; Kissler & Koessler, 2011; Delaney-Busch & Kuperberg, 2013; Rodríguez-Gómez et al., 2016). One study revealed N400 modulation by unconsciously perceived masked words as priming stimuli (Kiefer, 2002). Literature suggests that the N400 can also be elicited by non-semantic stimuli (e.g., Duncan et al., 2009; Yu et al., 2022), even though N400 effects might be smaller for non-linguistic stimuli (Yu et al., 2022; Tang et al., 2020). Considering most studies on the N400 still set a semantic framework even when introducing non-semantic stimuli to the study design (e.g. Aguado et al., 2013; Mori et al., 2021; Yu et al., 2022; Maquate et al., 2023), it is still unclear whether the N400 is sensitive to incongruencies in the context of a backward masking paradigm using emotional facial expressions as both prime and target stimulus.

The LPP is reflected by a positive peak starting at 200-300 ms lasting up to multiple seconds after stimulus onset (Cuthbert et al., 2000) and is associated with emotional perception (Liu et al., 2012; Brown et al., 2012). The LPP is reported to be sensitive to incongruency, with larger amplitudes in incongruent compared to congruent trials (Herring et al., 2011; Hinojosa et al., 2009; Diéguez-Risco et al., 2013; Morioka et al., 2016). However, reports of either reversed (Dozolme et al., 2015; Xu et al., 2017; Aguado et al., 2019; Huerta-Chavez & Ramos-Loyo, 2024) or null results can also be found (Aguado et al., 2013). The use of faces as stimuli causing a LPP response is well-established (e.g., Schupp et al., 2004b; Werheid et al., 2005; Bublatzky et al., 2014; Duval et al., 2013), with emotional facial expressions eliciting larger LPP amplitudes than control stimuli (Huerta-Chavez & Ramos-Loyo, 2024). However, to our knowledge, conclusive results on the effect of different primer presentation times as well as emotional incongruencies in the context of backward masking on the LPP have not been established yet.

A secondary aim is to examine the effect of emotional conflict created by trials with incongruent primer-target emotion. The N400 was robustly observed in response to incongruencies in the context of semantic primes such as words or sentences (for review see Kutas & Federmeier, 2011), but it is unclear if the component can also be observed when both primer and target stimulus consist of facial expressions. Applying the prevailing findings based on semantic stimuli (e.g., Zhang et al., 2006; Yu et al., 2022), we hypothesized that N400 amplitudes are larger in incongruent compared to congruent trials. In line with this, incongruent trials are expected to elicit larger LPP amplitudes than congruent trials (e.g., Hinojosa et al., 2009; Diéguez-Risco et al., 2013).

**Material and Methods**

**EEG Data processing**

For the analysis of the N400, channels were chosen at frontal (F3, Fz, F4), central (C3, Cz, C4) and parietal (P3, Pz, P4) sites (Duncan et al., 2009; Šoškić et al., 2022). Mean ERPs were calculated for each electrode site (frontal, central, parietal). For LPP analysis, the channels Fz, Cz and Pz were selected (Huerta-Chavez & Ramos-Loyo, 2024) and mean ERPs of the three channels were computed.

For the target analysis, N400 and LPP components were computed for every subject by averaging the segments for each target condition (happy, sad, and neutral target × unconscious and conscious primer presentation × primer-target emotion congruent and incongruent).

The preprocessed EEG data were segmented to epochs ranging from -200 to 1200 ms relative to the onset of the primer. The N400 was identified as the negative peak in the time interval ranging from 280 to 500 ms after target stimulus onset (Duncan et al., 2009; Šoškić et al., 2022). The LPP component was estimated as the positive peak between 500 to 700 ms after target onset (Huerta-Chavez & Ramos-Loyo, 2024).

**Statistical Analysis**

*Event-Related Potentials - LPP and N400*

For target analysis, N400 (Model 3) and LPP (Model 4) served as dependent variable in their respective model. Presentation time (mask; conscious or unconscious), emotion (target happy, sad, or neutral) and congruency (primer-target emotion congruent or incongruent) were set as fixed effects. A random intercept for each participant was included. Electrode site (location; frontal, central, or parietal) was included in the N400 model.

Model3 <- lmer(N400 ~ congruence + target emotion + mask + location + (1|participant)

Model4 <- lmer(LPP ~ congruence + target emotion + mask

+ (1|participant)

*Questionnaire Analysis - ERPs*

To test for possible relationships between different questionnaires and the ERP components (P100, N170, N400, LPP), multiple linear regression models were computed. The ERP amplitude served as the dependent variable in their respective regression model, different questionnaire scores were included as independent variables.

Model6 <- lm(P100 ~ TMT + BVAQ + MWTB + WMS forwards + WMS backwards, data = data)

Model7 <- lm(N170 ~ TMT + BVAQ + MWTB + WMS forwards + WMS backwards, data = data)

Model8 <- lm(N400 ~ TMT + BVAQ + MWTB + WMS forwards + WMS backwards, data = data)

Model9 <- lm(LPP ~ TMT + BVAQ + MWTB + WMS forwards + WMS backwards, data = data)

*Questionnaire Analysis - Accuracy*

Accounting for non-normality of accuracy data distribution, a generalized linear mixed model was computed using the Penalized Quasi-Likelihood method to test for possible correlations between questionnaire scores and accuracy. The accuracy value served as dependent variable while questionnaire scores were selected as independent variables. A random intercept for each participant was included.

Model10 <- glmmPQL(accuracy ~ TMT + BVAQ + MWTB + WMS forwards

+ WMS backwards, random = ~ 1|participant, family = quasipoisson(link = "log"), data = data)

# **Results**

## **Questionnaires**

### Event-Related Potentials

#### P100

For the P100, significant effects of the TMT, BVAQ, MWT-B and the WMS forwards were found (see Table S1). P100 amplitudes were greater in participants with higher TMT, MWT-B and WMS forwards scores compared to participants with lower scores in these questionnaires, while the effect of BVAQ scores showed opposite results.

**Table S1**

*Effect on P100 value: Type I Analysis of Variance Table for P100 Model.*

| Measure | *Sum Sq* | *Mean Sq* | *Df* | *F value* | *p value* |
| --- | --- | --- | --- | --- | --- |
| TMT (B/A Norm) | 107.7 | 107.73 | 1 | 7.44 | .007 ** |
| BVAQ (Sum) | 234.7 | 234.74 | 1 | 16.22 | < .001 *** |
| MWT-B (Norm) | 93 | 92.99 | 1 | 6.43 | .012 * |
| WMS forwards (Sum) | 787.8 | 787.78 | 1 | 54.44 | < .001 *** |
| WMS backwards (Sum) | 50.3 | 50.3 | 1 | 3.48 | .063 |

*Note*. BVAQ = Bermond-Vorst Alexithymia Questionnaire; MWT-B = Mehrfachwahl-Wortschatz-Intelligenztest (engl. multiple choice verbal intelligence test); TMT = Trail Making Test; WMS-R = Wechsler Memory Scale-Revised. “sum” = total score, “Norm” = normalized scale.

#### N170

For the N170 component, the model revealed significant effects of the MWT-B and the WMS backwards (see Table S2). N170 amplitudes were greater in participants with higher MWT-B and WMS backwards scores compared to participants with lower scores in the respective questionnaire.

**Table S2**

*Effect on N170 value: Type I Analysis of Variance Table for N170 Model.*

| Measure | *Sum Sq* | *Mean Sq* | *Df* | *F value* | *p value* |
| --- | --- | --- | --- | --- | --- |
| TMT (B/A Norm) | 0.40 | 0.41 | 1 | 0.03 | .854 |
| BVAQ (Sum) | 21.90 | 21.89 | 1 | 1.80 | .181 |
| MWT-B (Norm) | 66.40 | 66.43 | 1 | 5.45 | .020 * |
| WMS forwards (Sum) | 2.60 | 2.55 | 1 | 0.21 | .647 |
| WMS backwards (Sum) | 154.60 | 154.57 | 1 | 12.69 | < .001 *** |

*Note*. BVAQ = Bermond-Vorst Alexithymia Questionnaire; MWT-B = Mehrfachwahl-Wortschatz-Intelligenztest (engl. multiple choice verbal intelligence test); TMT = Trail Making Test; WMS-R = Wechsler Memory Scale-Revised. “sum” = total score, “Norm” = normalized scale.

#### N400

The linear regression model testing the effect of questionnaire scores on the N400 showed significant effects of the TMT, the BVAQ, the MWT-B and the WMS backwards (see Table S3). Larger N400 amplitudes were elicited in participants who had higher scores in the TMT, the MWTB and the WMS backward compared to participants who scored lower in these questionnaires, while the effect of the BVAQ showed opposite results.

**Table S3**

*Effect on N400 value: Type I Analysis of Variance Table for N400 Model.*

| Measure | *Sum Sq* | *Mean Sq* | *NumDF* | *F value* | *p value* |
| --- | --- | --- | --- | --- | --- |
| TMT (B/A Norm) | 139.90 | 139.91 | 1 | 22.35 | < .001 *** |
| BVAQ (Sum) | 64.40 | 64.42 | 1 | 10.29 | .001 ** |
| MWT-B (Norm) | 74.80 | 74.83 | 1 | 11.95 | < .001 *** |
| WMS forwards (Sum) | 5.00 | 5.00 | 1 | 0.80 | .372 |
| WMS backwards (Sum) | 86.00 | 86.00 | 1 | 13.74 | < .001 *** |

*Note*. BVAQ = Bermond-Vorst Alexithymia Questionnaire; MWT-B = Mehrfachwahl-Wortschatz-Intelligenztest (engl. multiple choice verbal intelligence test); TMT = Trail Making Test; WMS-R = Wechsler Memory Scale-Revised. “sum” = total score, “Norm” = normalized scale.

#### LPP

For the LPP component, significant effects of the TMT, the BVAQ and the WMS forwards were found (see Table S4). Larger LPP amplitudes were found in participants who received higher scores in the BVAQ and the WMS forwards compared to LPP amplitudes in participants with lower scores in the respective questionnaires, whereas the effect of the TMT scores showed opposite results.

**Table S4**

*Effect on LPP value: Type I Analysis of Variance Table for LPP Model.*

| Measure | *Sum Sq* | *Mean Sq* | *NumDF* | *F value* | *p value* |
| --- | --- | --- | --- | --- | --- |
| TMT (B/A Norm) | 75.32 | 75.32 | 1 | 30.69 | < .001 *** |
| BVAQ (Sum) | 208.03 | 208.03 | 1 | 84.77 | < .001 *** |
| MWT-B (Norm) | 8.63 | 8.63 | 1 | 3.52 | .061 |
| WMS forwards (Sum) | 45.57 | 45.57 | 1 | 18.57 | < .001 *** |
| WMS backwards (Sum) | 0.88 | 0.88 | 1 | 0.36 | .549 |

*Note*. BVAQ = Bermond-Vorst Alexithymia Questionnaire; MWT-B = Mehrfachwahl-Wortschatz-Intelligenztest (engl. multiple choice verbal intelligence test); TMT = Trail Making Test; WMS-R = Wechsler Memory Scale-Revised. “sum” = total score, “Norm” = normalized scale.

### Accuracy

The generalized linear mixed model revealed significant effects of the WMS forwards on the response accuracy. Accuracy scores were higher in participants with larger WMS forwards scores compared to participants with lower WMS forwards scores. No other questionnaire had a significant effect on the accuracy (see Table S5).

**Table S5**

*Effect on Accuracy value: Summary of Accuracy Model.*

| Measure | *Value* | *SD* | *NumDF* | *t value* | *p value* |
| --- | --- | --- | --- | --- | --- |
| TMT (B/A Norm) | < - 0.01 | 0.02 | 45 | - 0.03 | .975 |
| BVAQ (Sum) | < - 0.01 | < 0.01 | 45 | - 0.29 | .770 |
| MWT-B (Norm) | 0.05 | 0.03 | 45 | 1.81 | .077 |
| WMS forwards (Sum) | 0.03 | 0.01 | 45 | 2.57 | .014 * |
| WMS backwards (Sum) | - 0.01 | 0.01 | 45 | - 0.84 | .405 |

*Note*. BVAQ = Bermond-Vorst Alexithymia Questionnaire; MWT-B = Mehrfachwahl-Wortschatz-Intelligenztest (engl. multiple choice verbal intelligence test); TMT = Trail Making Test; WMS-R = Wechsler Memory Scale-Revised. “sum” = total score, “Norm” = normalized scale.

**Event-Related Potentials**

P100 Model Selection

For model selection, a model describing variance in P100 was tested with and without an interaction effect between primer emotion and the primer presentation time “mask”. An ANOVA was conducted to estimate model quality criteria for both models (Table S6). Model comparison for variance in P100 using Akaike Information Criterion (AIC) and Bayesian Information Criterion (BIC) did not indicate a significant improvement when adding an interaction effect between prime emotion and prime presentation time ("mask"). The model with interaction showed a slightly higher AIC and BIC (2754.0 and 2802.6, respectively) compared to the model without interaction (AIC = 2750.2, BIC = 2789.9). The likelihood ratio test yielded a Chi-square value of 0.2002 (df = 2, p = 0.9047), indicating that the addition of the interaction effect did not significantly enhance model fit.

**Table S6**

*ANOVA between models with and without interaction between primer emotion and primer presentation time (mask).*

| Model 1 | npar | AIC | BIC | logLik | Deviance | Chisq | Df | Pr(>Chisq) |
| --- | --- | --- | --- | --- | --- | --- | --- | --- |
| no interaction | 9 | 2750.2 | 2789.9 | -1366.1 | 2732.2 |  |  |  |
| with interaction | 11 | 2754.0 | 2802.6 | -1366.0 | 2732.0 | 0.2002 | 2 | 0.9047 |

*Note.* Model with no interaction: Model1 <- lmer(P100 ~ mask + primer emotion + sex + hemisphere + age + (1|participant)), Model with interaction: Model1 <- lmer(P100 ~ mask*primer emotion + sex + hemisphere + age + (1|participant)).

N170 Model Selection

For model selection, a model describing variance in N170 was tested with and without an interaction effect between prime emotion and the prime presentation time (“mask”). An ANOVA was conducted to compare model quality criteria for both models (Table S). Model comparison for variance in N170 using AIC BIC did not indicate a significant improvement when adding the interaction effect. The model with interaction showed a slightly higher AIC and BIC (2711.7 and 2760.3, respectively) compared to the model without interaction (AIC = 2709.2, BIC = 2748.9). The likelihood ratio test yielded a Chi-square value of 1.4346 (df = 2, p = 0.4881), suggesting that the addition of the interaction effect did not significantly enhance model fit.

**Table S7**

*ANOVA between models with and without interaction between prime emotion and prime presentation time (mask).*

| Model | npar | AIC | BIC | logLik | Deviance | Chisq | Df | Pr(>Chisq) |
| --- | --- | --- | --- | --- | --- | --- | --- | --- |
| No interaction | 9 | 2709.2 | 2748.9 | -1345.6 | 2691.2 |  |  |  |
| With interaction | 11 | 2711.7 | 2760.3 | -1344.9 | 2689.7 | 1.4346 | 2 | 0.4881 |

**Note:** Model with no interaction: Model2 <- lmer(N170 ~ mask + primer emotion + sex + hemisphere + age + (1|participant)). Model with interaction: Model2 <- lmer(N170 ~ mask*primer emotion + sex + hemisphere + age + (1|participant)).

N400

A significant effect of congruence (F(1,1779) = 15.57, p < .001) and scalp location was found (F(1,1779) = 346.21, p < .001) (Table S8). N400 was larger in congruent compared to incongruent trials (Figure S1A, Table S8). N400 amplitudes were largest on the frontal scalp (Figure S1D, Figure S4, Table S8).

**Table S8**

*Effect on N400 values: Type II Analysis of Variance Table with Kenward-Roger's method for N400 model.*

| Measure | *Sum Sq* | *Mean Sq* | *NumDF* | *df* | *F value* | *p value* |
| --- | --- | --- | --- | --- | --- | --- |
| congruence | 54.65 | 54.65 | 1 | 1779 | 15.57 | < .001 *** |
| target emotion | 2.98 | 1.49 | 2 | 1779 | 0.42 | .654 |
| mask | 4.64 | 4.64 | 1 | 1779 | 1.32 | .250 |
| location | 2430.31 | 1215.15 | 2 | 1779 | 346.21 | < .001 *** |


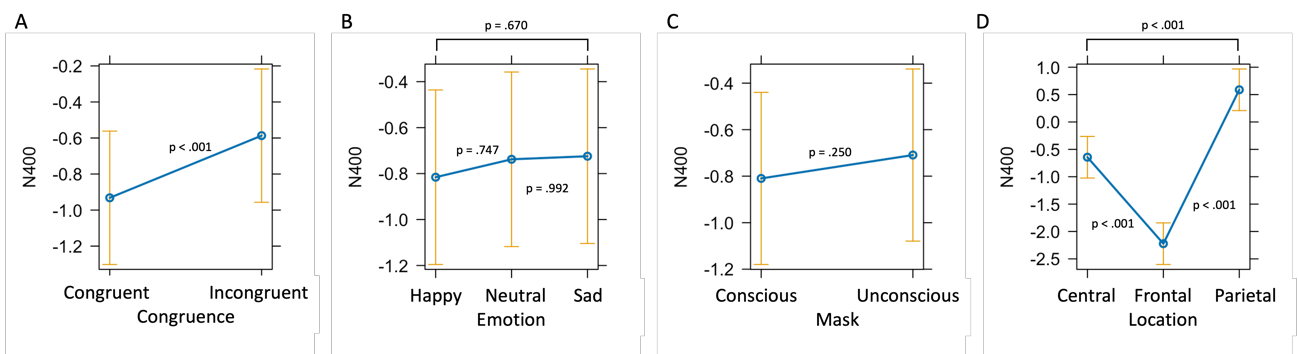


**Figure S1.** Visualization of parameter effects for N400 (estimated marginal means and standard error). Effect plot of (A) congruence, (B) emotion, (C) mask, (D) scalp topography location.

LPP

A significant effect of congruence was found (*F*(1,557) = 6.80, *p* = .009) (Table S9). Larger LPP amplitudes were found in congruent compared to incongruent trials (Figure S2A, Figure S3, Table S9).

**Table S9**

*Effect on LPP values: Type II Analysis of Variance Table with Kenward-Roger's method for LPP model.*

| Measure | *Sum Sq* | *Mean Sq* | *NumDF* | *df* | *F value* | *p value* |
| --- | --- | --- | --- | --- | --- | --- |
| congruence | 8.15 | 8.15 | 1 | 557 | 6.80 | .009 ** |
| target emotion | 4.06 | 2.03 | 2 | 557 | 1.69 | .185 |
| mask | 0.56 | 0.56 | 1 | 557 | 0.47 | .495 |


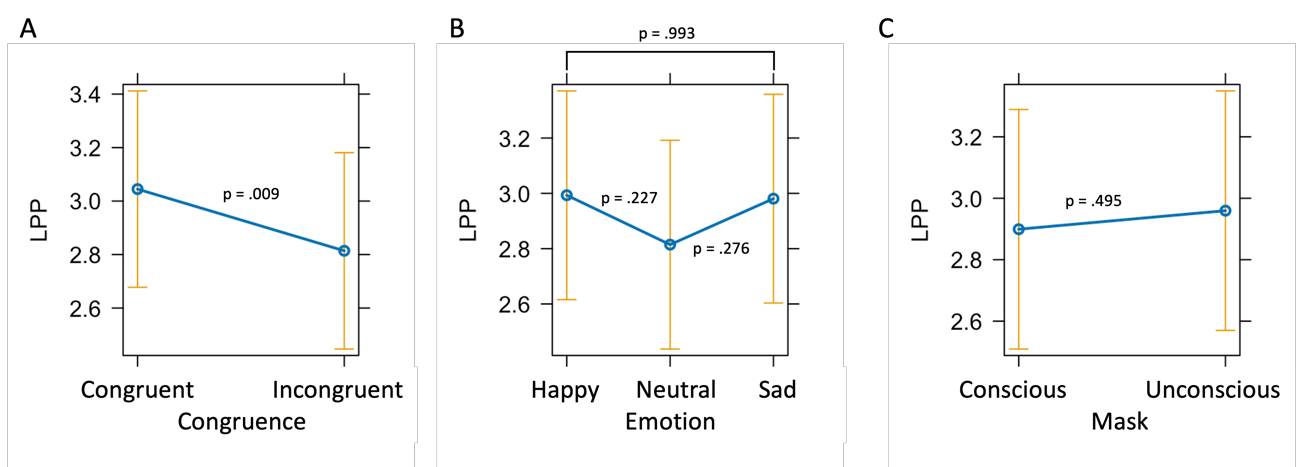


**Figure S2.** Visualization of parameter effects for LPP (estimated marginal means and standard error). Effect plot of (A) congruence, (B) emotion, (C) mask.


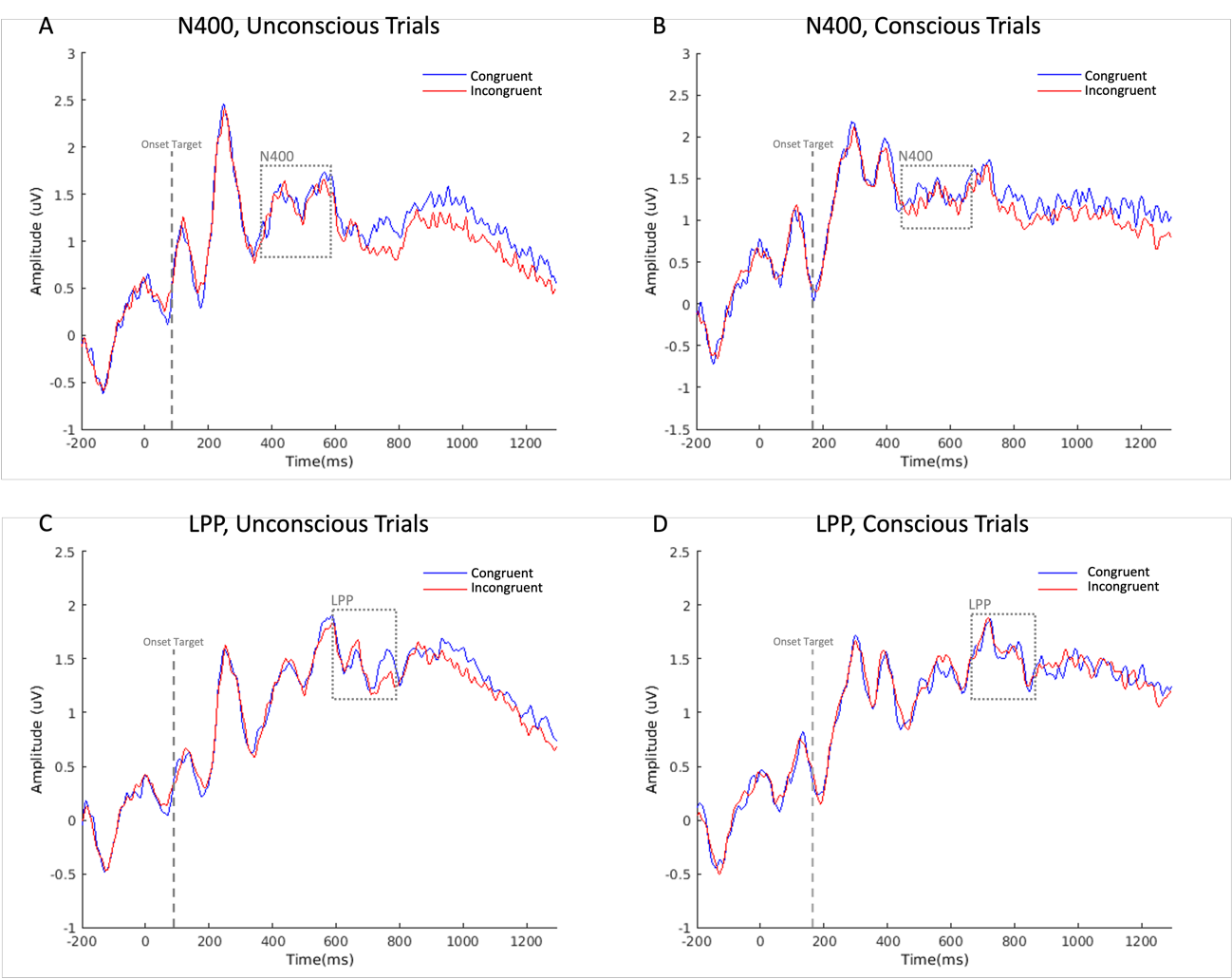


**Figure S3.** Mean N400 amplitudes at channels F3/Fz/F4/C3/Cz/C4/P3/Pz/P4 during (A) unconscious, (B) conscious trials. Mean LPP amplitudes at channels Fz/Cz/Pz during (C) unconscious, (D) conscious trials.


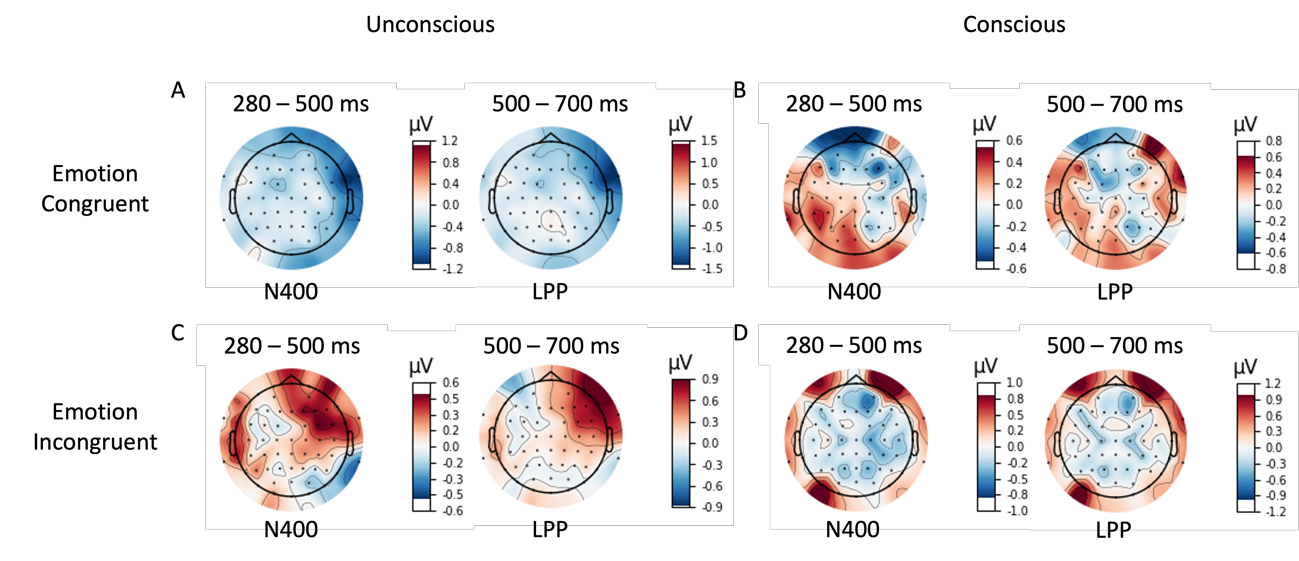


**Figure S4.** Mean Scalp Topography Maps for N400/LPP. Average amplitude in time interval 280-500 ms (N400) and 500-700 ms (LPP) post target stimulus. Unconscious trials with (A) congruent, (C) incongruent primer-target emotion. Conscious trials with (B) congruent, (D) incongruent primer-target emotion.

P100/N170

The complete analysis of both ERPs referring to the primer (P100/N170) can be found in the main manuscript.


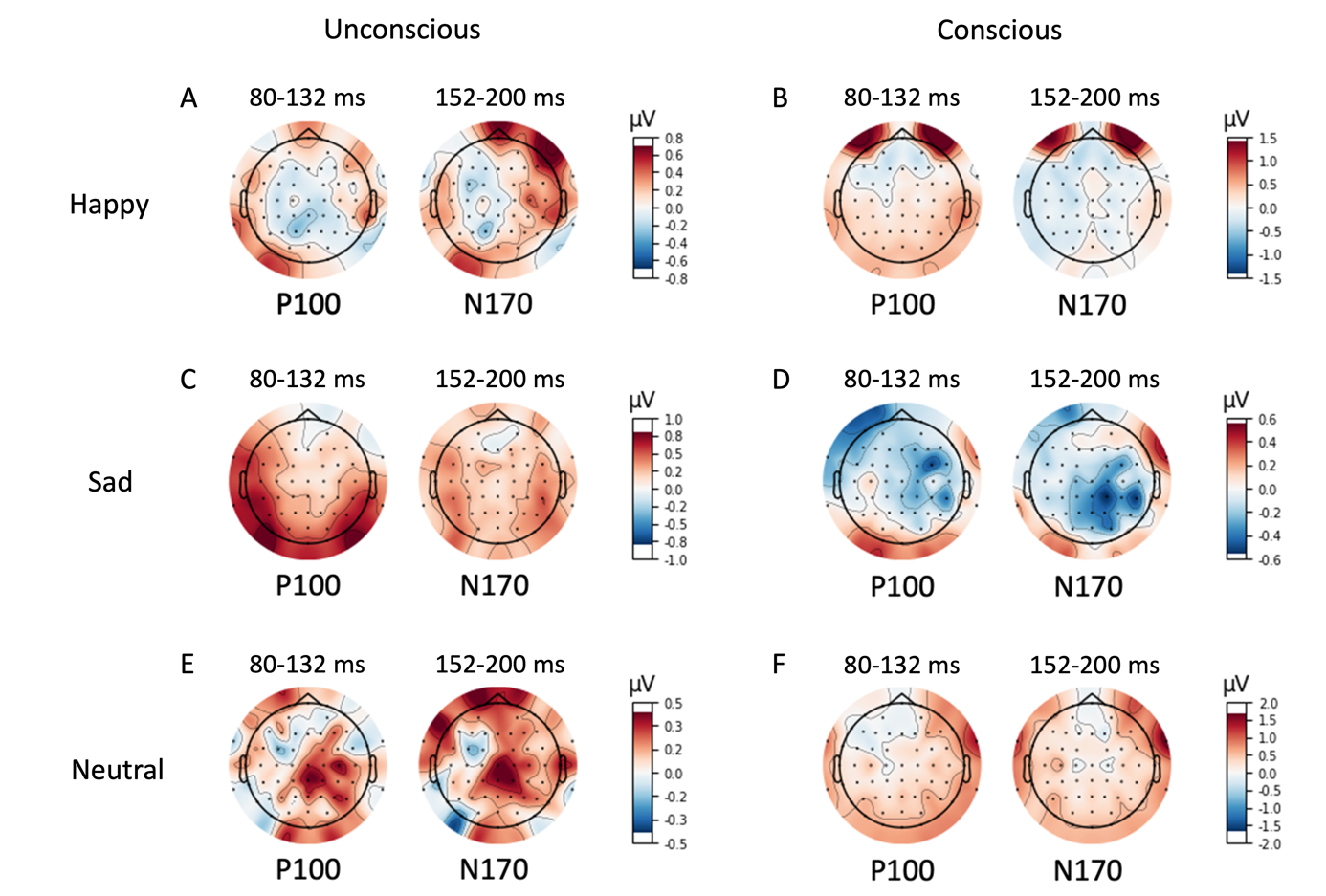


**Figure S5.** Mean Scalp Topography Maps for P100/N170. Average amplitude in time interval 80-132 ms (P100) and 152-200 ms (N170) post primer. Unconscious conditions with primer emotion (A) happy, (C) sad, (E) neutral. Conscious conditions with primer emotion (B) happy, (D) sad, (F) neutral.

**Discussion**

The N400 and LPP were used to examine the effect of incongruency between primer and target emotion. For both components, congruent trials elicited larger amplitudes than incongruent trials, which is contradictory to the hypothesis and the established function of the N400. Considering sensitivity to emotional incongruency, the N400/LPP seemed to reflect more complex cognitive processes. Unconscious primer presentation showed no effect on N400/LPP amplitudes.

*N400/LPP - emotional incongruence processing*

Typically, the N400 shows a broad scalp distribution (Kutas & Federmeier, 2011), showing largest amplitudes at midline central or parietal sites. However, when using pictures as stimuli, scalp distribution is reported to be more anterior (Duncan et al., 2009). This might explain our results of finding the largest N400 amplitudes at frontal sites. Some studies suggest the N400/LPP components might indicate violations of expectations (e.g. Kotz & Paulmann, 2007; Dozolme et al., 2015; Yu et al., 2022). We tested this functional modulation in our paradigm assuming larger effects in the components in trials where the primer emotion was followed by an incongruent target emotion. We did not observe the expected incongruency effect. One explanation may be that we did not induce sufficient expectancy violation as our emotions were presented in random orders. Still, the finding of larger N400 and LPP amplitudes in congruent compared to incongruent trials is contrary to the prevailing view in literature (for review see Kutas & Federmeier, 2011) and has to be further explored.

Considering that words elicited larger N400 effects than faces (Yu et al., 2022), the N400 is more suitable for researching semantic incongruencies rather than emotional facial expression incongruencies. The effects observed in this experiment may thus not be comparable to research findings on linguistic features.

Neither the N400 nor the LPP amplitude were found to be affected by conscious versus unconscious primer presentation time. One study, examining different conscious primer presentation times, reports larger N400 values in congruent versus incongruent trials with 200 ms primer presentation time, while a presentation time of 400 ms showed opposite results (Paulmann & Pell, 2010). This indicates that the N400 may be modulated by the stimulus presentation time but not whether a stimulus is processed consciously or unconsciously. Modulation of the LPP was found for both longer (1.5 s) as well as shorter (120 ms) stimulus presentation time (Schupp et al., 2000, 2004a). One study reported incongruency effects on LPP amplitudes by using a primer presentation time of 150 ms (Herring et al., 2011). Studies comparing different primer presentation times effects below the level of awareness on N400 and LPP are still lacking.

The lack of emotion effect on the N400/LPP is in contrast to several studies which reported modulation of amplitudes by different emotional stimuli (Schupp et al., 2004b; Cuthbert et al., 2000; Holt et al., 2009; Hajcak et al., 2006). This finding might be explained by differences in the study design by using a backward masking paradigm including facial expressions as both prime and target stimuli. Previous literature proposed that emotion processing might be a more automatic process, while congruency processing might be a more complex process that is reflected in everyday experience in which facial expressions are generally evaluated within context (Diéguez-Risco et al., 2013). Therefore, our findings reinforce the assumption that fast processing of emotions (reflected by emotion-, and timing-specific N170 and P100 values) and more complex cognitive processes such as evaluation of emotional congruency within context (reflected by congruency-specific N400 and LPP) might be separate cognitive processes operating at different times.

**Limitations**

The baseline correction for analysis of the N400/LPP was carried out 200 ms preceding the primer onset. Establishing a baseline correction directly before the target onset was not possible due to a short masking time. Therefore, using a larger masking time between primer and target stimuli, might lead to a more accurate result.

**References**

Aguado, L., Dieguez-Risco, T., Méndez-Bértolo, C., Pozo, M. A., & Hinojosa, J. A. (2013). Priming effects on the N400 in the affective priming paradigm with facial expressions of emotion. *Cognitive, affective & behavioral neuroscience*, *13*(2), 284–296. https://doi.org/10.3758/s13415-012-0137-3

Aguado, L., Parkington, K. B., Dieguez-Risco, T., Hinojosa, J. A., & Itier, R. J. (2019). Joint Modulation of Facial Expression Processing by Contextual Congruency and Task Demands. *Brain sciences*, *9*(5), 116. https://doi.org/10.3390/brainsci9050116

Brown, S. B., van Steenbergen, H., Band, G. P., de Rover, M., & Nieuwenhuis, S. (2012). Functional significance of the emotion-related late positive potential. *Frontiers in human neuroscience*, *6*, 33. https://doi.org/10.3389/fnhum.2012.00033

Bublatzky, F., Gerdes, A. B., White, A. J., Riemer, M., & Alpers, G. W. (2014). Social and emotional relevance in face processing: happy faces of future interaction partners enhance the late positive potential. *Frontiers in human neuroscience*, *8*, 493. https://doi.org/10.3389/fnhum.2014.00493

Cuthbert, B. N., Schupp, H. T., Bradley, M. M., Birbaumer, N., & Lang, P. J. (2000). Brain potentials in affective picture processing: covariation with autonomic arousal and affective report. *Biological psychology*, *52*(2), 95–111. https://doi.org/10.1016/s0301-0511(99)00044-7

Delaney-Busch, N., & Kuperberg, G. (2013). Friendly drug-dealers and terrifying puppies: affective primacy can attenuate the N400 effect in emotional discourse contexts. *Cognitive, affective & behavioral neuroscience*, *13*(3), 473–490. https://doi.org/10.3758/s13415-013-0159-5

Diéguez-Risco, T., Aguado, L., Albert, J., & Hinojosa, J. A. (2013). Faces in context: modulation of expression processing by situational information. *Social neuroscience*, *8*(6), 601–620. https://doi.org/10.1080/17470919.2013.834842

Dozolme, D., Brunet-Gouet, E., Passerieux, C., & Amorim, M. A. (2015). Neuroelectric Correlates of Pragmatic Emotional Incongruence Processing: Empathy Matters. *PloS one*, *10*(6), e0129770. https://doi.org/10.1371/journal.pone.0129770

Duncan, C. C., Barry, R. J., Connolly, J. F., Fischer, C., Michie, P. T., Näätänen, R., Polich, J., Reinvang, I., & Van Petten, C. (2009). Event-related potentials in clinical research: guidelines for eliciting, recording, and quantifying mismatch negativity, P300, and N400. *Clinical neurophysiology : official journal of the International Federation of Clinical Neurophysiology*, *120*(11), 1883–1908. https://doi.org/10.1016/j.clinph.2009.07.045

Duval, E. R., Moser, J. S., Huppert, J. D., & Simons, R. F. (2013). What’s in a face? The late positive potential reflects the level of facial affect expression. *Journal of Psychophysiology, 27*(1), 27–38. https://doi.org/10.1027/0269-8803/a000083

Eder, A. B., Leuthold, H., Rothermund, K., & Schweinberger, S. R. (2012). Automatic response activation in sequential affective priming: an ERP study. *Social cognitive and affective neuroscience*, *7*(4), 436–445. https://doi.org/10.1093/scan/nsr033

Hajcak, G., Moser, J. S., & Simons, R. F. (2006). Attending to affect: appraisal strategies modulate the electrocortical response to arousing pictures. *Emotion (Washington, D.C.)*, *6*(3), 517–522. https://doi.org/10.1037/1528-3542.6.3.517

Herring, D. R., Taylor, J. H., White, K. R., & Crites, S. L., Jr. (2011). Electrophysiological responses to evaluative priming: The LPP is sensitive to incongruity. Emotion, 11(4), 794–806. https://doi.org/10.1037/a0022804

Hietanen, J. K., & Astikainen, P. (2013). N170 response to facial expressions is modulated by the affective congruency between the emotional expression and preceding affective picture. *Biological psychology*, *92*(2), 114–124. https://doi.org/10.1016/j.biopsycho.2012.10.005

Hinojosa, J. A., Carretié, L., Méndez-Bértolo, C., Míguez, A., & Pozo, M. A. (2009). Arousal contributions to affective priming: electrophysiological correlates. *Emotion (Washington, D.C.)*, *9*(2), 164–171. https://doi.org/10.1037/a0014680

Holt, D. J., Lynn, S. K., & Kuperberg, G. R. (2009). Neurophysiological correlates of comprehending emotional meaning in context. *Journal of cognitive neuroscience*, *21*(11), 2245–2262. https://doi.org/10.1162/jocn.2008.21151

Huerta-Chavez, V., & Ramos-Loyo, J. (2024). Emotional congruency between faces and words benefits emotional judgments in women: An event-related potential study. *Neuroscience letters*, *822*, 137644. https://doi.org/10.1016/j.neulet.2024.137644

Kiefer M. (2002). The N400 is modulated by unconsciously perceived masked words: further evidence for an automatic spreading activation account of N400 priming effects. *Brain research. Cognitive brain research*, *13*(1), 27–39. https://doi.org/10.1016/s0926-6410(01)00085-4

Kissler, J., & Koessler, S. (2011). Emotionally positive stimuli facilitate lexical decisions—An ERP study. *Biological psychology*, *86*(3), 254-264.

Kotz, S. A., & Paulmann, S. (2007). When emotional prosody and semantics dance cheek to cheek: ERP evidence. *Brain research*, *1151*, 107–118. https://doi.org/10.1016/j.brainres.2007.03.015

Kutas, M., & Federmeier, K. D. (2011). Thirty years and counting: finding meaning in the N400 component of the event-related brain potential (ERP). Annual review of psychology, 62, 621–647. https://doi.org/10.1146/annurev.psych.093008.131123

Kutas, M., & Hillyard, S. A. (1980). Reading senseless sentences: Brain potentials reflect semantic incongruity. Science, 207(4427), 203-205.

Liu, Y., Huang, H., McGinnis-Deweese, M., Keil, A., & Ding, M. (2012). Neural substrate of the late positive potential in emotional processing. *The Journal of neuroscience : the official journal of the Society for Neuroscience*, *32*(42), 14563–14572. https://doi.org/10.1523/JNEUROSCI.3109-12.2012

Maquate, K., Kissler, J., & Knoeferle, P. (2023). Speakers' emotional facial expressions modulate subsequent multi-modal language processing: ERP evidence. *Language, Cognition and Neuroscience*, *38*(10), 1492-1513.

Mori, K., Tanaka, A., Kawabata, H., & Arao, H. (2021). The N400 and late occipital positivity in processing dynamic facial expressions with natural emotional voice. *NeuroReport: For Rapid Communication of Neuroscience Research, 32*(10), 858–863. https://doi.org/10.1097/WNR.0000000000001669

Morioka, S., Osumi, M., Shiotani, M., Nobusako, S., Maeoka, H., Okada, Y., Hiyamizu, M., & Matsuo, A. (2016). Incongruence between Verbal and Non-Verbal Information Enhances the Late Positive Potential. *PloS one*, *11*(10), e0164633. https://doi.org/10.1371/journal.pone.0164633

Morris, J. P., Squires, N. K., Taber, C. S., & Lodge, M. (2003). Activation of Political Attitudes: A Psychophysiological Examination of the Hot Cognition Hypothesis. *Political Psychology, 24*(4), 727–745. https://doi.org/10.1046/j.1467-9221.2003.00349.x

Paulmann, S., & Pell, M. D. (2010). Contextual influences of emotional speech prosody on face processing: how much is enough?. *Cognitive, affective & behavioral neuroscience*, *10*(2), 230–242. https://doi.org/10.3758/CABN.10.2.230

Rodríguez-Gómez, P., Sánchez-Carmona, A., Smith, C., Pozo, M. A., Hinojosa, J. A., & Moreno, E. M. (2016). On the violation of causal, emotional, and locative inferences: An event-related potentials study. *Neuropsychologia*, *87*, 25–34. https://doi.org/10.1016/j.neuropsychologia.2016.04.032

Schauenburg, G., Conrad, M., von Scheve, C., Barber, H. A., Ambrasat, J., Aryani, A., & Schröder, T. (2019). Making sense of social interaction: Emotional coherence drives semantic integration as assessed by event-related potentials. *Neuropsychologia*, *125*, 1–13. https://doi.org/10.1016/j.neuropsychologia.2019.01.002

Schupp, H. T., Cuthbert, B. N., Bradley, M. M., Cacioppo, J. T., Ito, T., & Lang, P. J. (2000). Affective picture processing: the late positive potential is modulated by motivational relevance. *Psychophysiology*, *37*(2), 257–261.

Schupp, H. T., Junghöfer, M., Weike, A. I., & Hamm, A. O. (2004a). The selective processing of briefly presented affective pictures: an ERP analysis. *Psychophysiology*, *41*(3), 441–449. https://doi.org/10.1111/j.1469-8986.2004.00174.x

Schupp, H. T., Ohman, A., Junghöfer, M., Weike, A. I., Stockburger, J., & Hamm, A. O. (2004b). The facilitated processing of threatening faces: an ERP analysis. *Emotion (Washington, D.C.)*, *4*(2), 189–200. https://doi.org/10.1037/1528-3542.4.2.189

Šoškić, A., Jovanović, V., Styles, S. J., Kappenman, E. S., & Ković, V. (2022). How to do Better N400 Studies: Reproducibility, Consistency and Adherence to Research Standards in the Existing Literature. *Neuropsychology review*, *32*(3), 577–600. https://doi.org/10.1007/s11065-021-09513-4

Steinbeis, N., & Koelsch, S. (2011). Affective priming effects of musical sounds on the processing of word meaning. *Journal of cognitive neuroscience*, *23*(3), 604–621. https://doi.org/10.1162/jocn.2009.21383

Tang, M., Chen, B., Zhao, X., & Zhao, L. (2020). Processing network emojis in Chinese sentence context: An ERP study. *Neuroscience letters*, *722*, 134815. https://doi.org/10.1016/j.neulet.2020.134815

Werheid, K., Alpay, G., Jentzsch, I., & Sommer, W. (2005). Priming emotional facial expressions as evidenced by event-related brain potentials. *International journal of psychophysiology : official journal of the International Organization of Psychophysiology*, *55*(2), 209–219. https://doi.org/10.1016/j.ijpsycho.2004.07.006

Xu, Q., Yang, Y., Tan, Q., & Zhang, L. (2017). Facial Expressions in Context: Electrophysiological Correlates of the Emotional Congruency of Facial Expressions and Background Scenes. *Frontiers in psychology*, *8*, 2175. https://doi.org/10.3389/fpsyg.2017.02175

Yu, L., Xu, Q., Cao, F., Liu, J., Zheng, J., Yang, Y., & Zhang, L. (2022). Emotional violation of faces, emojis, and words: Evidence from N400. Biological psychology, 173, 108405. https://doi.org/10.1016/j.biopsycho.2022.108405

Zhang, Q., Lawson, A., Guo, C., & Jiang, Y. (2006). Electrophysiological correlates of visual affective priming. *Brain research bulletin*, *71*(1-3), 316–323. https://doi.org/10.1016/j.brainresbull.2006.09.023
